# PM_10_ Impairs CD56^dim^ NK Cell Cytotoxicity via FNBP1 Suppression to Exacerbate Rheumatoid Arthritis: Insights from Multimodal Multi‐Omics

**DOI:** 10.1002/advs.202514260

**Published:** 2026-02-20

**Authors:** Runhan Zhao, Qinyang Zhang, Yu Jiang, Dagang Tang, Xiao Qu, Jun Zhang, Yi Chen, Weixia Duan, Tao Li, Zhengwei Cai, Yanran Huang, Xiaoji Luo

**Affiliations:** ^1^ Department of Orthopaedic Chongqing Municipal Health Commission Key Laboratory of Musculoskeletal Regeneration and Translational Medicine/Orthopaedic Research Laboratory The First Affiliated Hospital of Chongqing Medical University Chongging P. R. China; ^2^ School of Public Health Chongqing Medical University Chongging P. R. China; ^3^ Department of Orthopaedics Shanghai Key Laboratory for Prevention and Treatment of Bone and Joint Diseases Shanghai Institute of Traumatology and Orthopaedics, Ruijin Hospital Shanghai Jiao Tong University School of Medicine Shanghai P. R. China; ^4^ Chongqing Municipal Health Commission Key Laboratory for Emergency Poisoning Detection and Acute Care The First Affiliated Hospital of Chongqing Medical and Pharmaceutical College Chongging P. R. China; ^5^ Department of Orthopaedic Chongqing General Hospital Chongqing University Chongging P. R. China; ^6^ Department of Orthopedics The First Affiliated Hospital of Chongqing Medical and Pharmaceutical College Chongging

**Keywords:** formin binding protein 1, natural killer cells, particulate matter, PM_10_, rheumatoid arthritis

## Abstract

Air pollution (AP), intensified by industrialization and urbanization, is a key environmental factor linked to rheumatoid arthritis (RA). However, its molecular and immunological impact on RA remains unclear. This study integrates epidemiological data, bioinformatics, single‐cell transcriptomics, and animal models to investigate how AP contributes to the development of RA. Global epidemiological analysis shows rising RA prevalence in over 95% of countries. Mendelian randomization analysis indicated a positive correlation between PM_10_ exposure and the risk of RA. Machine learning identifies Formin Binding Protein 1 (FNBP1) as a key air pollution‐related gene (APRG), with decreasing expression in RA patients and strong correlation with disease activity. PM_10_ exposure may impair natural killer (NK) cell differentiation and cytotoxicity by suppressing FNBP1 expression, ultimately weakening immune surveillance and exacerbating inflammatory responses. Furthermore, by integrating single‐cell sequencing, animal models, and human‐derived cell experiments, we demonstrated that PM_10_ exposure aggravates inflammation and joint damage in a collagen‐induced arthritis (CIA) model. Mechanistically, PM_10_ likely impairs the cytotoxic function of CD56^dim^ NK cells through the modulation of FNBP1. Taken together, our research results have unveiled a completely novel mechanistic hypothesis regarding the onset and development of RA, the “PM_10_‐FNBP1‐NK cells” axis.

## Graphical abstract

This study, through multi‐omics approaches and animal models, revealed that air pollutant PM_10_ exacerbates the progression of rheumatoid arthritis (RA) by suppressing FNBP1 expression and impairing the cytotoxic function of CD56^dim^ NK cells. The “PM_10–_FNBP1–NK cells” axis provided novel insights into the environmental pathogenesis of RA and suggested FNBP1 as a potential therapeutic target.

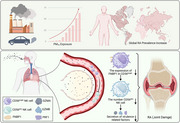

## 1 Introduction

Amid the global wave of industrialization and the rapid acceleration of urbanization, pollution sources such as industrial emissions and vehicle exhaust continuously release large quantities of particulate matter (PM_2.5_ and PM_10_) and toxic gases, including sulfur dioxide and nitrogen oxides, into the atmosphere [1, 2]. According to data from the World Health Organization, more than 90% of the global population has been chronically exposed to environments with poor air quality [3]. Air pollution (AP) not only directly threatens the respiratory system but can also penetrate the alveolar‐capillary barrier to enter the bloodstream, thereby inducing systemic pathological responses [4, 5]. These responses, in particular, affect immune function, contributing to the development of cardiovascular diseases, neurological disorders, musculoskeletal conditions, and a range of autoimmune diseases [6, 7]. The effects of environmental pollutants extend well beyond localized respiratory damage, as they can disrupt molecular signaling pathways and impair normal cellular functions, leading to immune dysregulation [8]. Given the immune system's pivotal role in defending against external pathogens, its impairment provides a critical foundation for the onset and progression of chronic illnesses, posing a significant threat to human health.

Rheumatoid arthritis (RA) is a systemic autoimmune disorder primarily characterized by chronic synovial inflammation and progressive joint destruction [9]. Although its etiology remains incompletely understood, epidemiological studies have consistently shown that prolonged exposure to AP significantly increases the risk of RA development [10, 11]. The underlying biological mechanisms linking AP to RA, however, are yet to be fully elucidated. Current evidence suggests that inhaled pollutants provoke localized inflammation and oxidative stress in the lungs, which can activate the immune system and disrupt immune tolerance [12, 13]. Notably, Natural Killer (NK) cells—critical mediators of immune surveillance and self‐tolerance—appear to play a pivotal role in this process [14]. Dysfunction of NK cells may contribute to the initiation of RA in its early stages.

In the intricate regulatory network governing immune cell function, Formin Binding Protein 1 (FNBP1) has emerged as a central molecular hub [15]. As a member of the F‐BAR domain protein family, FNBP1 features an N‐terminal extended FER‐CIP4 homology (EFC) domain, which efficiently induces plasma membrane deformation, and a C‐terminal Src homology 3 (SH3) domain [16]. The SH3 domain mediates the recruitment of Wiskott–Aldrich syndrome protein (WASP), WASP‐interacting protein (WIP), and Dynamin‐2 to the plasma membrane, thereby facilitating actin polymerization [17]. A Rho‐binding domain (RBD) located between the EFC and SH3 domains enables interaction with Rho family GTPases, contributing to downstream signal transduction [18]. FNBP1 plays an essential role in cell migration, immune synapse formation, and endocytosis, and is particularly critical for the dynamic functional regulation of innate immune cells [19].

Continuous exposure to environmental pollutants markedly suppresses the expression of FNBP1 in both peripheral blood and the tissue microenvironment [20]. In NK cells, this downregulation triggers aberrant subpopulation programming: the differentiation of CD56^bright^ NK cells into their cytotoxic CD56^dim^ counterparts is hindered, resulting in a pronounced reduction in the expression and secretion of cytotoxic effector molecules [21, 22]. As key sentinels of the innate immune system, NK cells play a crucial role in eliminating senescent, transformed, or aberrantly activated cells [23]. Impairment of their function compromises immune surveillance and disrupts the fundamentals of immune homeostasis [24]. The collapse of this surveillance mechanism establishes a pathological foundation for aberrant autoimmune activation and chronic inflammation [25].

**SCHEME 1:**
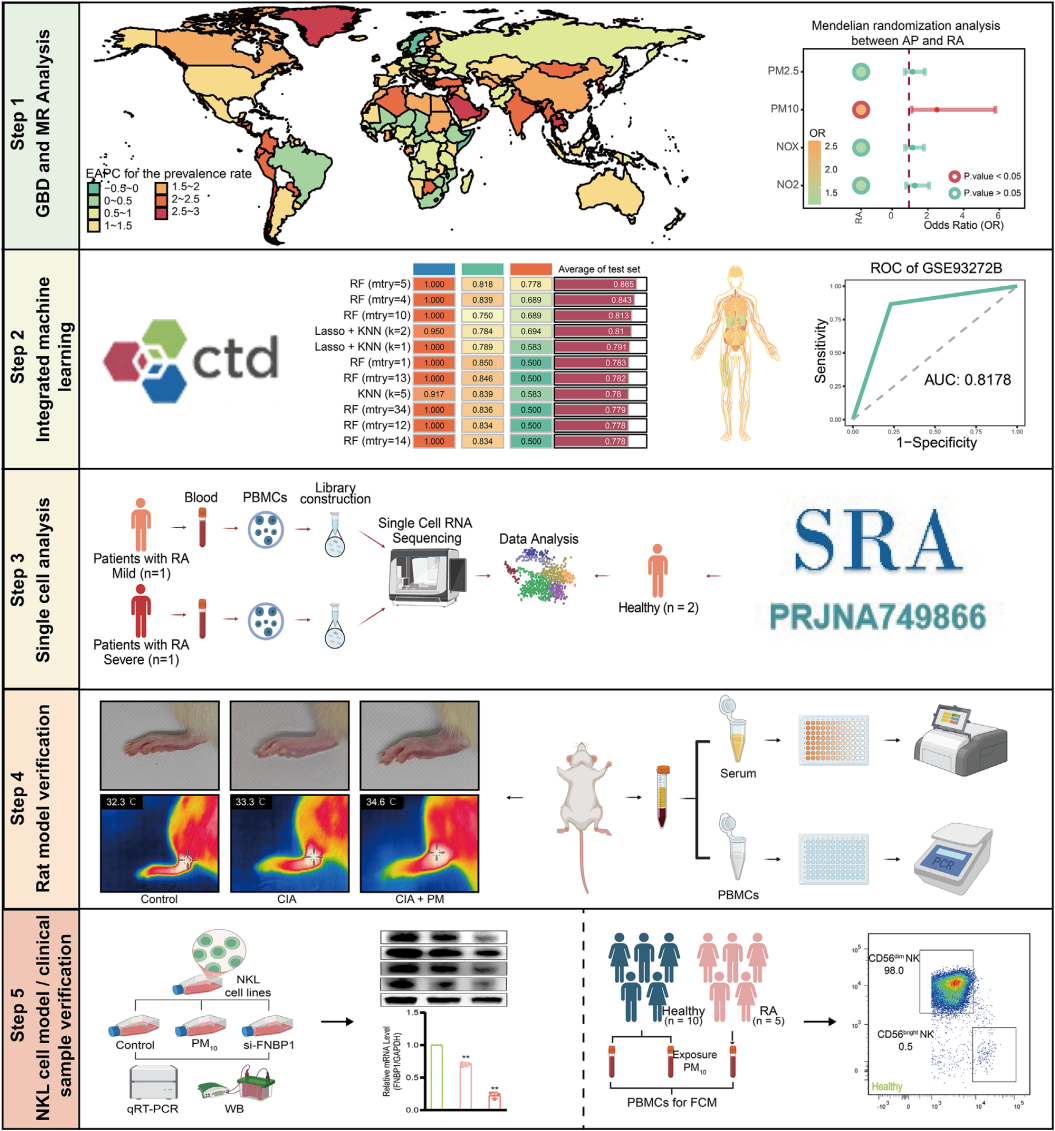
Schematic illustration of technical roadmap.

Ultimately, the collapse of immune homeostasis meticulously triggered by environmental pollutants at the molecular and cellular levels culminates in a destructive cascade within the joint cavity. Dysfunctional immune cells infiltrate the synovium, unleashing a storm of pro‐inflammatory factors, driving abnormal synovial hyperplasia, and forming invasive pannus that erodes cartilage and bone—hallmarks of the chronic synovitis and progressive joint destruction characteristic of RA. Environmental toxins disrupt immune balance by suppressing the expression of FNBP1, leading to the functional inactivation of NK cells, a critical early event that primes the joint for destructive inflammation. This study employed multi‐omics approaches to delineate the PM10^–^FNBP1^–^NK cell axis (Scheme 1). Elucidating the specific molecular and cellular mechanisms through PM_10_ exposure induces RA via the FNBP1–NK cell axis not only addresses a critical knowledge gap between environmental exposure and autoimmune pathogenesis but also provides essential scientific evidence for the early identification of at‐risk populations, the formulation of targeted environmental intervention strategies, and the development of novel therapeutics.

## 2 Materials and Methods

### 2.1 Data Acquisition and Preprocessing

A list of air pollution‐related genes (APRGs) was obtained from the Comparative Toxicogenomics Database (CTD, https://ctdbase.org/, Table S1). Peripheral blood transcriptome data from patients with RA and healthy controls, as well as transcriptome data from peripheral blood mononuclear cells (PBMCs) exposed to particulate matter (PM), were retrieved from the Gene Expression Omnibus (GEO, https://www.ncbi.nlm.nih.gov/geo/). Detailed dataset information is provided in Table S2. Single‐cell RNA sequencing (scRNA‐seq) data from healthy individuals were accessed via the Sequence Read Archive (SRA; https://www.ncbi.nlm.nih.gov/sra) [26]. Additionally, with approval from our institutional ethics committee, venous blood samples were collected from two RA patients. PBMCs were isolated and submitted to BGI for single‐cell sequencing. Epidemiological data on RA (1990–2021) were obtained from the Global Burden of Disease (GBD) database, which includes detailed methodological and statistical information [27, 28]. Transcriptomic data across various human tissues were sourced from the Genotype‐Tissue Expression (GTEx) Project (https://www.genome.gov/Funded‐Programs‐Projects/Genotype‐Tissue‐Expression‐Project). Finally, genome‐wide association study (GWAS) data related to AP and RA were extracted from the published literature and are presented in Table S2 [29, 30].

#### 2.1.1 Transcriptome Data Preprocessing

Probe annotation was performed using the official annotation file. In cases where multiple probes mapped to the same gene, the probe with the highest average expression value was retained. Following annotation, the expression matrix was normalized using the limma R package. The processed data were then used for downstream analyses. Single‐Cell Data Preprocessing: Cell filtering was conducted using the Seurat R package (V 5.1.0). The inclusion criteria required that each gene be expressed in at least three cells and that each cell expresses more than 200 genes. Additionally, cells with mitochondrial gene expression exceeding 10% were excluded from the analysis. Data normalization was performed using the SCTransform (SCT) algorithm, and batch effects were corrected using the canonical correlation analysis (CCA) method. The resulting dataset was then used for subsequent analyses.

### 2.2 GBD Analysis

Based on the Global Burden of Disease (GBD) epidemiological data for RA, we calculated the estimated annual percentage change (EAPC) to assess trends in prevalence between 1990 and 2021.

Percentchange=(2021cases−1990cases)/1990cases

The EAPC is a widely recognized and effective metric frequently used in epidemiological studies to assess temporal trends in indicators, such as disease prevalence and incidence [31]. EAPC is calculated by fitting a regression model to the natural logarithm of the rate over time. Specifically, a linear regression is performed with the calendar year (x) as the independent variable and the natural logarithm of the observed rate (y) as the dependent variable. In this model, α denotes the intercept, β the slope, and ε the random error term. The EAPC is then derived from the estimated slope of this fitted line.

y=α+βx+ε

$$\mathrm{EAPC}\;=100\;\times \;\left(\exp\left(\beta \right)\;-\;1\right)$$

### 2.3 Mendelian Randomization Analysis of AP and RA

Based on the three core assumptions of Mendelian randomization — (1) the relevance assumption, wherein instrumental variables (IVs) are strongly associated with the exposure of interest; (2) the independence assumption, which states that IVs are not associated with confounding factors; and (3) the exclusion restriction assumption, indicating that IVs influence the outcome solely through the exposure pathway [32] we selected instrumental single nucleotide polymorphisms (SNPs) according to the following thresholds: p < 5 × 10^−^^6^, r^2^ < 0.01, F > 10 and a window size of 10 000 kb. Mendelian randomization (MR) analysis was then performed, using inverse variance weighting (IVW) as the primary analytical method. When the *p* value of the consistency test is less than 0.05, the IVW random effects model (IVW‐MRE) is employed. Additional methods, including MR‐Egger, simple mode, weighted median, and weighted mode, were employed as complementary sensitivity approaches. Finally, robustness of the results was assessed using sensitivity analyses based on Cochran's Q test, MR‐Egger intercept test, and the leave‐one‐out approach.

### 2.4 Utilizing Integrated Machine Learning to Screen for Key Genes

Initially, univariate logistic regression was applied to reduce the dimensionality of APRGs in each dataset, and candidate key genes were identified by intersecting the results. Subsequently, key gene selection was refined through an integrated machine learning approach involving 15 algorithms: least absolute shrinkage and selection operator (LASSO), neural network‐multilayer Perceptron (NN‐MLP), logistic regression (LR), linear discriminant analysis (LDA), quadratic discriminant analysis (QDA), k‐nearest neighbors (KNN), decision trees (DT), random forests (RF), XGBoost, ridge regression (RR), elastic net regression (ENR), support vector machines (SVM), gradient boosting machine (GBM), stepwise logistic regression (S‐LR), and Naive Bayes. The integration process proceeded as follows: first, a model was trained using the optimal parameters identified via the caret package; second, feature selection was refined using the LASSO algorithm, followed by retraining the model under the same parameter optimization procedure; finally, models were ranked based on their performance using the area under the receiver operating characteristic curve (ROC) to identify the best predictive model. Key genes were ultimately determined by referencing the feature importance rankings across more than 200 machine learning models.

### 2.5 Preliminary Exploration of the Functions of Key Genetic Molecules

Based on GTEx data, the expression profiles of key genes across various tissues were analyzed, with particular emphasis on peripheral blood. Concurrently, clinical relevance was assessed by generating ROC curves and performing correlation analyses between gene expression and key clinical characteristics using the available dataset. To further validate the findings, transcriptomic data from PBMCs exposed to PM_10_ were analyzed for differential gene expression, aiming to determine whether the identified key genes exhibited similar expression patterns in both RA and PM_10_ exposure. The immunoregulatory roles of these genes were further explored through gene set enrichment analysis (GSEA) and immune cell infiltration analysis. Additionally, the association between immune cell abundance and RA disease severity was examined using the Spearman correlation coefficient. Finally, molecular docking simulations were conducted to evaluate the binding affinities and potential targeting effects of PM_10_ on the identified key genes.

Molecular Docking: First, the 3D structures of the candidate compounds and key target proteins were obtained from the PubChem (https://pubchem.ncbi.nlm.nih.gov) and Protein Data Bank (PDB; http://www.rcsb.org) databases. The target protein structures were then preprocessed by removing water molecules and co‐crystallized ligands, adding hydrogen atoms, and repairing any missing side chains to optimize structural integrity. Molecular docking simulations were performed using AutoDock (V 1.5.6) to assess the binding interactions between the compounds and target proteins. The optimal docking conformations were subsequently visualized using PyMOL (V 3.1) and Discovery Studio (2019).

### 2.6 Exploring the Immune Regulatory Functions of Key Genes Based on Single‐Cell Data

Following dimensionality reduction of the cells, key immune cell markers were employed for cell annotation and subsequent in‐depth analysis. First, the expression levels of critical genes across different samples and cell types were examined to validate their pivotal roles in RA. Next, functional alterations in key regulatory immune cells were confirmed through cell communication analysis and GSEA. Subsequently, key regulatory immune cell subsets were extracted, subjected to further dimensionality reduction and clustering, and re‐annotated. The newly defined subsets were then verified and their proportions assessed. Thereafter, expression changes of key genes and principal cytokines involved in immune regulation in RA were evaluated, accompanied by correlation analyses. Additionally, pseudotime analysis was performed to investigate the roles of key genes in the differentiation of immune cells. The pseudotime analysis in this study was mainly accomplished using the ‘monocle3’ R package. During the data preprocessing stage, the num_dim parameter was set to 30, the cluster_method parameter was set to “louvain”, and all other parameters were set to their default values. Additionally, based on previous studies [33, 34], the root node was set to the CD56^bright^ NK cell subset. Finally, genes closely associated with the key genes were identified from the STRING database and subjected to Gene Ontology (GO) and Kyoto Encyclopedia of Genes and Genomes (KEGG) enrichment analyses to elucidate their molecular biological functions.

### 2.7 Quantitative Reverse Transcription PCR (qRT‐PCR)

Total RNA was extracted from rat blood using TRIzol reagent (Servicebio, Wuhan, China), following the manufacturer's instructions. Quantitative reverse transcription PCR (qRT‐PCR) was conducted on a LightCycler 480 system (Roche, Basel, Switzerland) using reverse transcription and qPCR kits (YiFeiXue Biotechnology). The PCR cycling conditions were as follows: initial denaturation at 95 ℃ for 2 min, followed by 36 cycles of denaturation at 95 ℃ for 15 s, annealing at 60 ℃ for 30 s, and extension at 72  ℃for 30 s. A final extension step at 72  ℃ for 30 s was included to complete fragment elongation. Primer sequences are listed in Table S3.

### 2.8 Cell Culture

The human natural killer (NK) cell line NKL (Shanghai Jingfeng Biotechnology Co., Ltd.) was cultured in RPMI 1640 medium (Gibco) supplemented with 10% fetal bovine serum (FBS; Gibco), 100 U/mL penicillin, and 100 µg/mL streptomycin (Gibco). Cells were maintained in a humidified incubator at 37°C with 5% CO_2_ and subcultured every 2–3 days to maintain a cell density of 0.5–2 × 10^6^ cells/mL.

### 2.9 NKL Transfection

NKL cells in the logarithmic growth phase were seeded into 6‐well plates at a density of 2–5 × 10^5^ cells/mL in antibiotic‐free RPMI‐1640 complete medium. siRNAs and Lipofectamine RNAiMAX (Thermo Fisher Scientific, Cat#13778030) were separately diluted in Opti‐MEM, mixed gently, and incubated at room temperature for 20 min. The siRNA–lipid complexes were then added dropwise to the cultures and mixed thoroughly. Cells were incubated at 37°C with 5% CO_2_ for 24–48 h. After transfection, cells were harvested for qRT‐PCR analysis of FNBP1 mRNA expression. Relative expression levels were calculated using the ΔΔCt method, and the siRNA with the highest silencing efficiency was selected for subsequent experiments (Figure S1). Three siRNAs targeting different regions of FNBP1 (siRNA‐FNBP1‐1, siRNA‐FNBP1‐2, and siRNA‐FNBP1‐3) and a negative control (si‐NC) were used in the experimental design.

Three siRNA oligonucleotides targeting different regions of FNBP1 were designed and synthesized (Tsingke Biotechnology, China). The sequences were as follows: siRNA‐FNBP1‐1: sense 5′‐UUUACUAGAUUCAAGCUGC‐3′, antisense 5′‐GCAGCUUGAAUCUAGUAAA‐3′; siRNA‐FNBP1‐2: sense 5′‐UUCUGAUCAAUUGAUUCGG‐3′, antisense 5′‐CCGAAUCAAUUGAUCAGAA‐3′; siRNA‐FNBP1‐3: sense 5′‐UUCAGUUCCUGAACAUAGC‐3′, antisense 5′‐GCUAUGUUCAGGAACUGAA‐3′.

### 2.10 Flow Cytometry

PBMC were isolated from fresh peripheral blood of healthy donors and patients with RA using density gradient centrifugation. For PM_10_ exposure, PBMCs from healthy donors were treated with 100µg/mL PM_10_ for 24h. Cells were then resuspended in FACS buffer (PBS containing 2% serum) and incubated with fluorescently conjugated monoclonal antibodies against human CD3, CD56, and CD16 for 30 min at 4°C in the dark. Following staining, cells were washed twice with FACS buffer and resuspended in 500 µL of FACS buffer for immediate flow cytometric analysis. The gating and analysis strategy is illustrated in Figure S2. In this study, the collection of human peripheral blood samples was approved by the Ethics Committee of the First Affiliated Hospital of Chongqing Medical University (Approval No. 2024‐080‐01).

### 2.11 Western Blot (WB)

Add the sample to be tested to the RIPA lysis buffer containing protease/phosphatase inhibitors and incubate it on ice. Centrifuge at 12000 rpm for 15 minutes at 4°C to collect the supernatant total protein. Then, perform gel electrophoresis and transfer the protein to a PVDF membrane activated by methanol in a wet transfer method. Run the membrane at a constant current of 400 mA for 40 minutes. After transfer, incubate with 5% skimmed milk at room temperature for 1 hour, add the primary antibody and incubate at 4°C overnight, wash with TBST three times, and then add the HRP‐labeled secondary antibody and incubate at room temperature for 1 hour. Color the bands using the ECL chemiluminescence method. Quantify the gray values of the bands using ImageJ software. The experiment should have at least 3 independent biological and technical replicates. Antibodies used in WB: GZMA (1:1000, 11288‐1‐AP, Proteintech), GZMB (1:1000, AF0175, Affinity), PRF1 (1:1000, DF6004, Affinity), FNBP1 (1:1000, DF15733, Affinity), and β‐actin (1:1000, 66009‐1‐Ig, Proteintech).

### 2.12 Animal Experiments

The animal experimental protocol was reviewed and approved by the Ethics Committee of Chongqing Medical University (Approval No. IACUC‐CQMU‐2025‐06078). All experiments were conducted in strict accordance with the Declaration of Helsinki and relevant Chinese regulations on the welfare and ethics of laboratory animals.

#### 2.12.1 PM_10_ Tracheal Instillation Mode

The test animals were anesthetized, and their tongues were gently extended using tweezers. Cotton swabs were used to absorb saliva in the oral cavity to prevent visual obstruction. The tongue was held with fingers, and a tongue depressor was inserted to expose and observe the opening and closing of the glottis. The30 µL suspension of PM_10_ was slowly administered directly to the glottis. Following instillation, 500 µL of air was gently introduced through the nostril using a needleless syringe to facilitate uniform distribution of the suspension. Successful administration was indicated by the presence of an audible respiratory sound, akin to a cough or expectoration, when the animal was held near the ear.

#### 2.12.2 Collagen‐Induced Arthritis (CIA) Model Establishment

SD Rats were housed in a controlled environment under a 12‐h light/dark cycle at 20–26 ℃ with 40%–70% relative humidity (n = 5 per cage). The CIA model was established by subcutaneous injection of type II bovine collagen (2 mg/mL) emulsified in an equal volume of complete Freund's adjuvant (CFA) at the base of the tail. On day 7, a booster injection of type II bovine collagen (2 mg/mL) emulsified with incomplete Freund's adjuvant was administered. Arthritis severity was assessed every three days by two independent observers using the following scoring criteria: 1 = erythema and mild swelling confined to the tarsal bones or ankle joint; 2 = erythema and mild swelling extending from the ankle joint to the tarsus; 3 = erythema and moderate swelling extending to the metatarsal joint; and 4 = erythema and severe swelling involving the ankle, claws, toes, or limb stiffness. On day 35, rats were anesthetized with pentobarbital and euthanized via cervical dislocation. Peripheral blood was collected for subsequent analysis.

#### 2.12.3 Infra‐Red Thermograph

The body surface temperature of rats was recorded using a FLIR infrared thermal imager (FLIR Systems, USA). Before imaging, the device was preheated and calibrated, with the emissivity set to 0.98 to align with the thermal properties of rat skin. All measurements were conducted under controlled ambient conditions (22°C–24°C) to minimize external thermal interference. To reduce motion artifacts without inducing stress, the rats were gently restrained in a transparent acrylic chamber. The thermal imager was positioned approximately 20 cm above the animals for vertical imaging. Thermal images were captured once the animals had reached a stable physiological state. All thermal images and associated temperature data were archived for subsequent quantitative analysis.

#### 2.12.4 Enzyme‐Linked Immunosorbent Assay (ELISA)

Peripheral blood was collected from rats centrifuged at 4°C. The resulting supernatant was stored at −80°C until further analysis. The concentrations of GZMA, GZMB, PRF1, and FNBP1 in the supernatant were quantified using enzyme‐linked immunosorbent assay kits (Jiangsu Meimian industrial Co., Ltd). Absorbance was measured at 450 nm using a microplate reader (BioTek, Vermont, USA).

#### 2.12.5 Sample Collection and Processing

On the 35th day, the rats were anesthetized with pentobarbital and then sacrificed. Peripheral blood was collected via cardiac puncture, and serum was separated by centrifugation at 4°C for subsequent ELISA and qRT‐PCR analyses.

### 2.13 Statistical Analysis

Transcriptomic data were normalized and transformed using the limma package with log transformation, and outlier samples were removed based on clustering algorithms. Single‐cell data were normalized using the SCTransform algorithm of the Seurat package, with batch effects corrected via the CCA method. Continuous variables are presented as mean ± standard deviation (mean ± SD). Multi‐group comparisons were performed using one‐way ANOVA. Correlation analyses were conducted using the Spearman method. All statistical analyses were performed using the R language (V 4.3.2) or GraphPad Prism (V 10.4.2). Unless otherwise specified, the significance level was set at *p* < 0.05.

## 3 Results

### 3.1 AP is a Critical Environmental Factor Contributing to the Pathogenesis of RA

**FIGURE 1:**
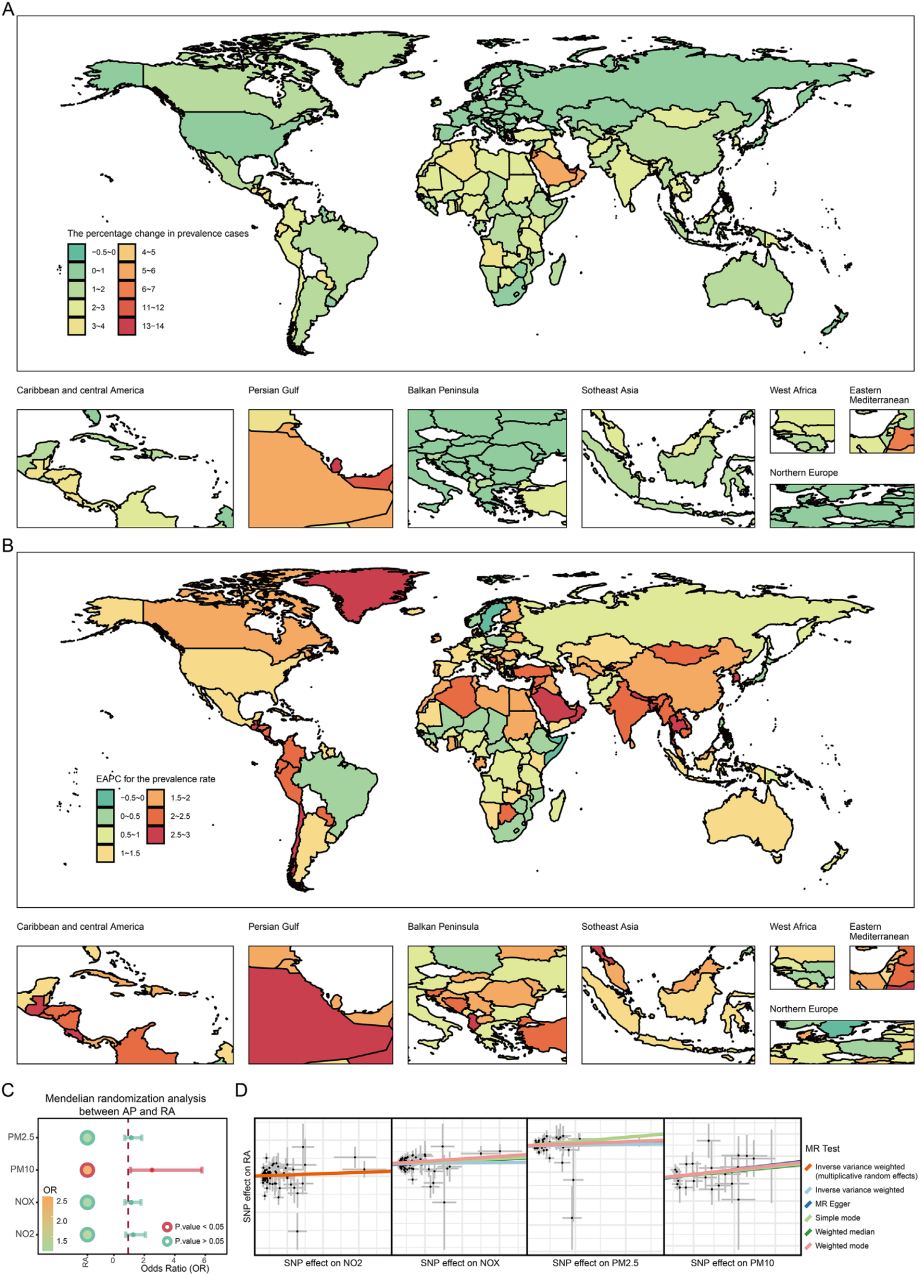
Global trends in the burden of RA. (A): Percentage change in the number of prevalent RA cases across 204 countries between 1990 and 2021 (RA, rheumatoid arthritis). (B): EAPC in RA prevalence rates across 204 countries from 1990 to 2021 (EAPC, estimated annual percentage change). (C): Mendelian randomization forest plot showing the associations between air pollutants and RA. (D): Mendelian randomization scatter plot depicting the causal relationship between air pollution exposure and RA.

Epidemiological data from the GBD study indicates a worsening trend in the prevalence of RA in over 95% of countries between 1990 and 2021. The most pronounced increases were observed in Albania (EAPC: 2.93, 95% CI: 2.82–3.04) and the Republic of Korea (EAPC: 2.89, 95% CI: 2.71–3.07) (Figure 1A,B; Table S4). With rapid economic development, industrial and vehicular emissions have markedly exacerbated air pollution. Mendelian randomization analysis revealed a positive association between exposure to AP particulate matter and RA risk, with PM_10_ showing a statistically significant effect (Figure 1C,D). These findings were further supported by sensitivity analyses (Figure S3 and Table S5), confirming their robustness. Collectively, these results suggest a significant causal relationship between air pollution and RA. The ongoing intensification of air pollution may be contributing to the rising global burden of RA, underscoring the urgent need for public health intervention and environmental regulation.

### 3.2 FNBP1 is a Key APRG Involved in RA

**FIGURE 2:**
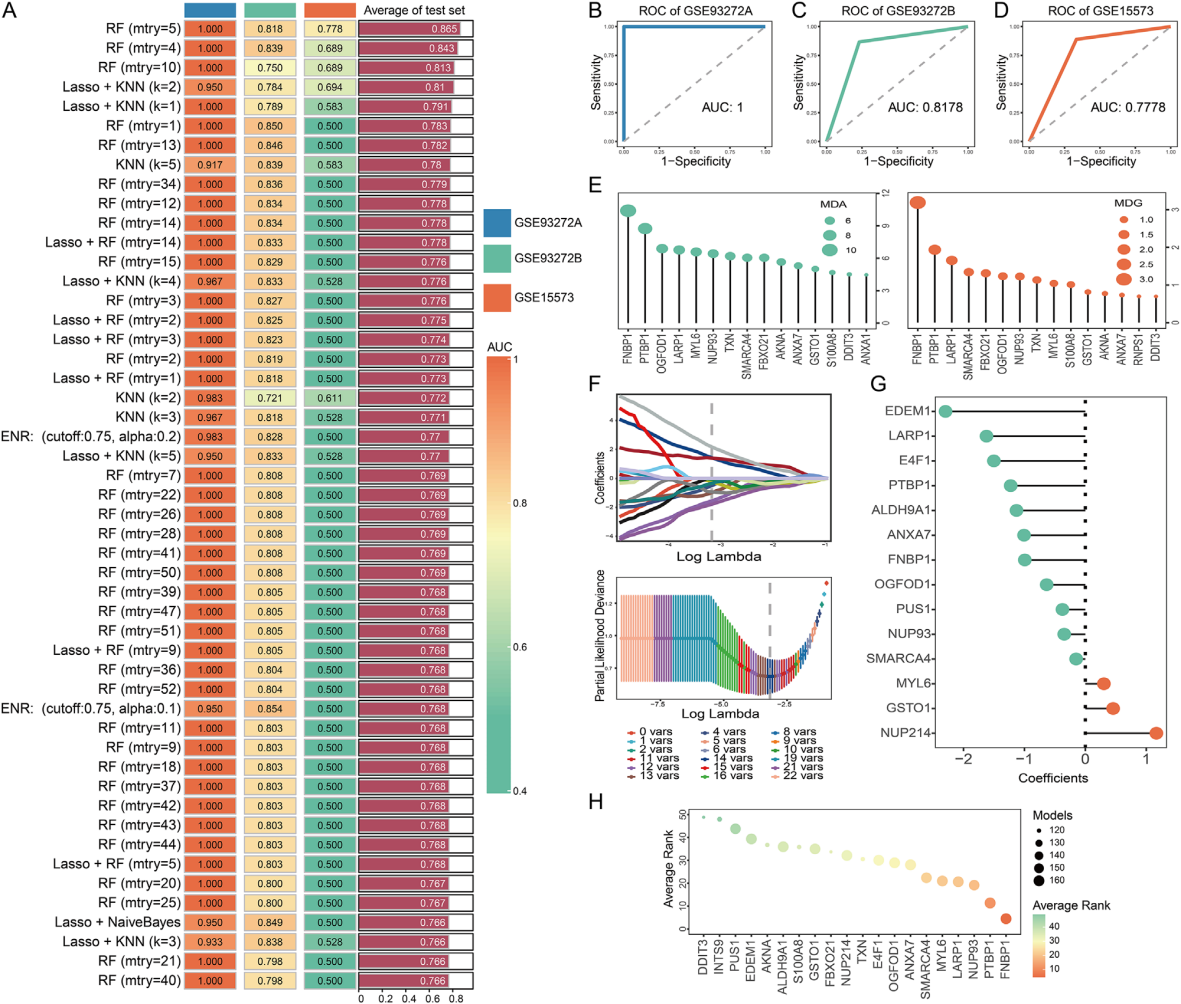
Identification of key APRGs using integrated machine learning approaches. (A): AUC heat map of the top 50 models ranked by predictive accuracy (AUC, Area Under the Curve). (B–D): ROC curves of the RF model (mtry = 5) across three independent datasets (ROC, receiver operating characteristic curve; RF, random forests). (E): Gene rankings based on MDA and MDG in the RF model (MDA, mean decrease accuracy; MDG, mean decrease gini). (F): Coefficient path plot and cross‐validation curve from the Lasso regression model. (G): Gene coefficient distribution derived from the Lasso algorithm. (H): Gene importance scores plotted using integrated machine learning algorithms. (nGSE93272A = 60, nGSE93272B = 215, and nGSE15573 = 33).

Using univariate logistic regression analysis, we identified 58 candidate key genes that were consistently significant across all datasets (Table S6). By integrating multiple machine learning algorithms, we constructed a total of 292 predictive models (Table S7). Among these, the random forest model with parameter setting mtry = 5 exhibited the most robust predictive performance, achieving Area Under the Curve (AUC) values greater than 0.75 across all three datasets (Figure 2A–D). Variable importance in the random forest models was assessed using mean decrease accuracy (MDA) and mean decrease gini (MDG), where higher values indicate greater importance. Notably, FNBP1 and PTBP1 ranked among the top two variables according to both MDA and MDG metrics (Figure 2E). To further evaluate the relevance of the 58 candidate genes, we applied the LASSO algorithm, which also identified FNBP1 and PTBP1 as key predictors (Figure 2F,G). Finally, out of the models that provided variable importance metrics (*n* = 84), over 50 models ranked FNBP1 and PTBP1 within the top two positions, with FNBP1 consistently emerging as the most influential feature Figure 2H).

### 3.3 FNBP1 Exhibits Substantial Clinical Relevance

**FIGURE 3:**
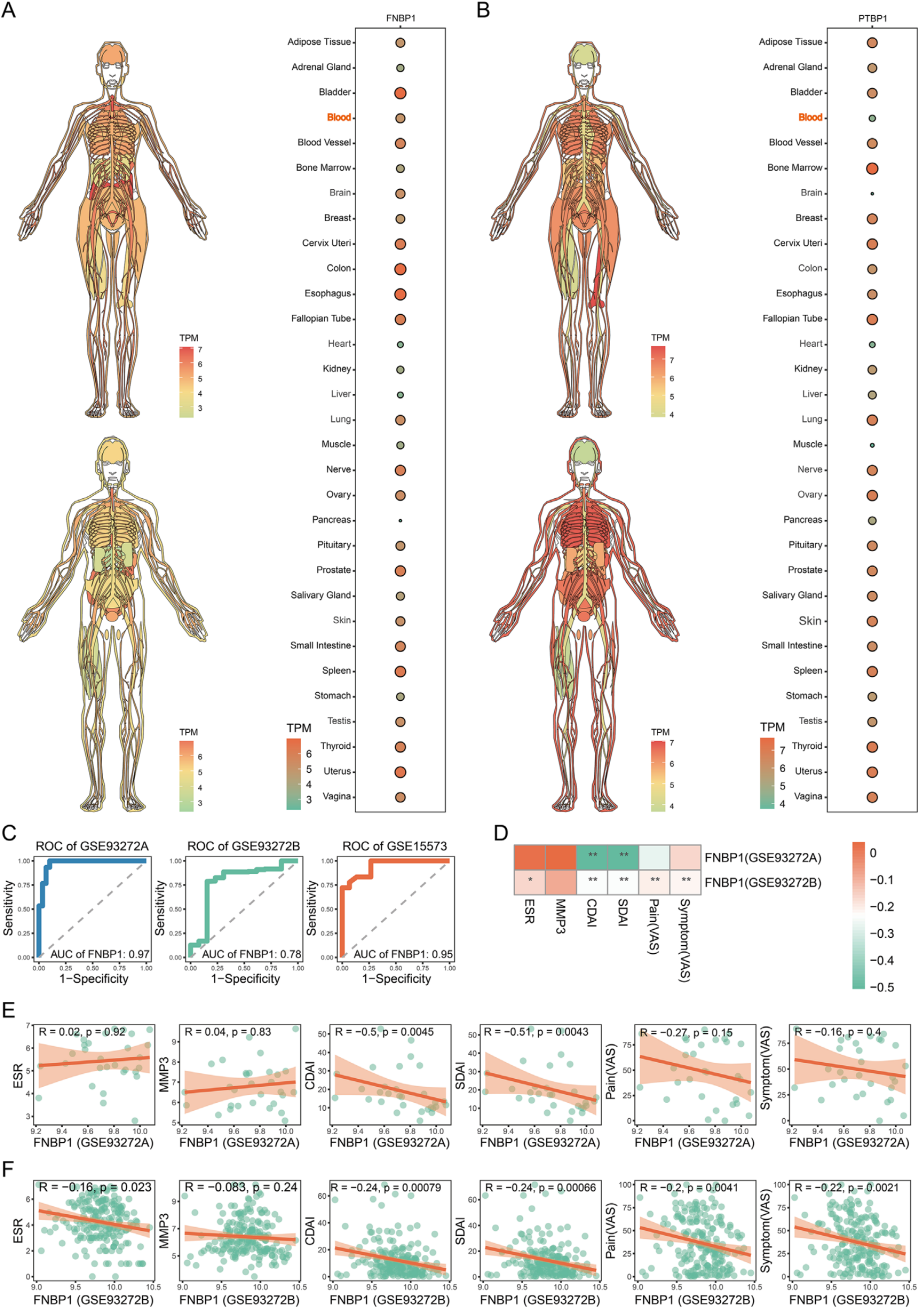
Clinical relevance of key APRGs. (A,B): Expression levels of FNBP1 and PTBP1 across various tissues in male and female subjects. (C): ROC curves assessing the predictive performance of FNBP1 expression for RA risk across three independent datasets. (D): Heatmap illustrating the correlation between FNBP1 expression and clinical features. (E,F): Scatter plots showing the relationship between FNBP1 expression and clinical features in the GSE93272A and GSE93272B cohorts. (^*^ *p* < 0.05, ^**^ *p* < 0.01; nGSE93272A = 60, nGSE93272B = 215, and nGSE15573 = 33).

In the analysis of gene expression across human tissues, we focused on the enrichment of key APRGs in peripheral blood, given its minimally invasive collection procedure and widespread clinical availability. Our findings revealed that FNBP1 exhibited significantly higher expression levels in blood compared to PTBP1 (Figure 3A,B), underscoring its superior potential for clinical applications. Moreover, FNBP1 demonstrated robust predictive performance across all three independent datasets (Figure 3C). Correlation analysis further reveals that in both the GSE93272A and GSE93272B datasets, FNBP1 expression levels exhibit a negative association with rheumatoid arthritis (RA) disease activity. Specifically, FNBP1 expression inversely correlates with the Clinical Disease Activity Index (CDAI) and the Simplified Disease Activity Index (SDAI). Moreover, assessments using the Visual Analogue Scale (VAS) indicate a significant negative correlation between FNBP1 expression and patient‐reported pain intensity (Figure 3D–F; Table S8). Collectively, these results highlight the strong clinical relevance of FNBP1 and its utility in both risk prediction and symptom evaluation in rheumatoid arthritis.

### 3.4 PM10 Alters FNBP1 Expression Levels and Contributes to the Impaired Cytotoxic Function of NK Cells

**FIGURE 4:**
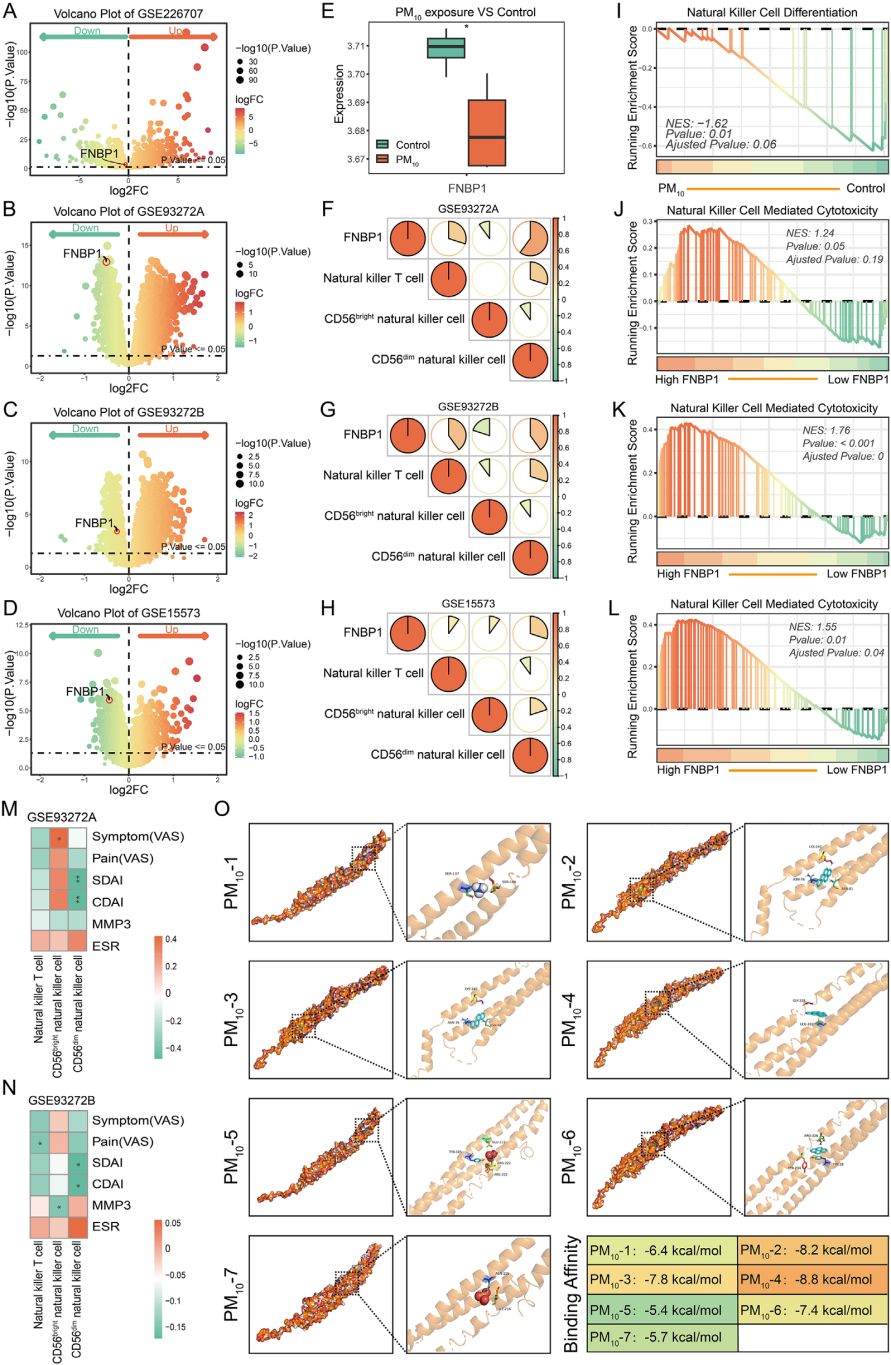
Preliminary investigation of the biological functions of FNBP1. (A–D): Volcano plots illustrating differentially expressed genes. (E): Box plot showing differential expression of FNBP1 between the PM_10_ exposure group and the control group. (F–H): Heatmaps depicting the correlation between FNBP1 expression and immune cell infiltration in datasets GSE93272A (F), GSE93272B (G), and GSE15573 (H). (I–L): GSEA based on PM_10_ exposure status (±) and FNBP1 expression levels (high/low). (M,N): Heatmap showing the correlation between NK cell infiltration and RA disease severity. (O): Molecular docking simulations of PM_10_ representative compounds with FNBP1. (^*^ *p* < 0.05, ^**^ *p* < 0.01; nGSE226707 = 8, nGSE93272A = 60, nGSE93272B = 215, and nGSE15573 = 33).

In the peripheral blood transcriptome data of PBMCs exposed to PM_10_ and RA, we observed a significant decrease in FNBP1 expression (Figure 4 A‐E). This finding supports the view that PM_10_‐induced downregulation of FNBP1 may represent a mechanism linking environmental exposure to the onset and progression of RA. Immune cell infiltration analysis further revealed that FNBP1 expression was positively correlated with the abundance of CD56^dim^ NK cells (Figure 4F–H; Table S8), with the higher FNBP1 levels the higher abundance of CD56^dim^ NK cells(Figure S4). GSEA analysis indicated that PM_10_ exposure suppresses the NK cells differentiation pathway (Figure 4I). Moreover, diminished FNBP1 expression was associated with impaired cytotoxic function of NK cells (Figure 4J–L). Previous studies have demonstrated that the cytotoxic activity of CD56^dim^ NK cells contributes to RA remission [35]. Consistently, our data showed a significant negative correlation between CD56^dim^ NK cell counts and RA disease activity (Figure 4M,N; Table S8). Finally, molecular docking simulations revealed that representative small molecules of PM_10_, including ammonium, benzo[a]pyrene, chrysene, indeno[1,2,3‐cd]pyrene, nitrate, pyrene, and sulfate, can directly bind to the molecular pocket of FNBP1 (Figure 4O; Table S9). This suggested a theoretical interaction between particulate matter and FNBP1, though further evidence is required for validation.

**FIGURE 5:**
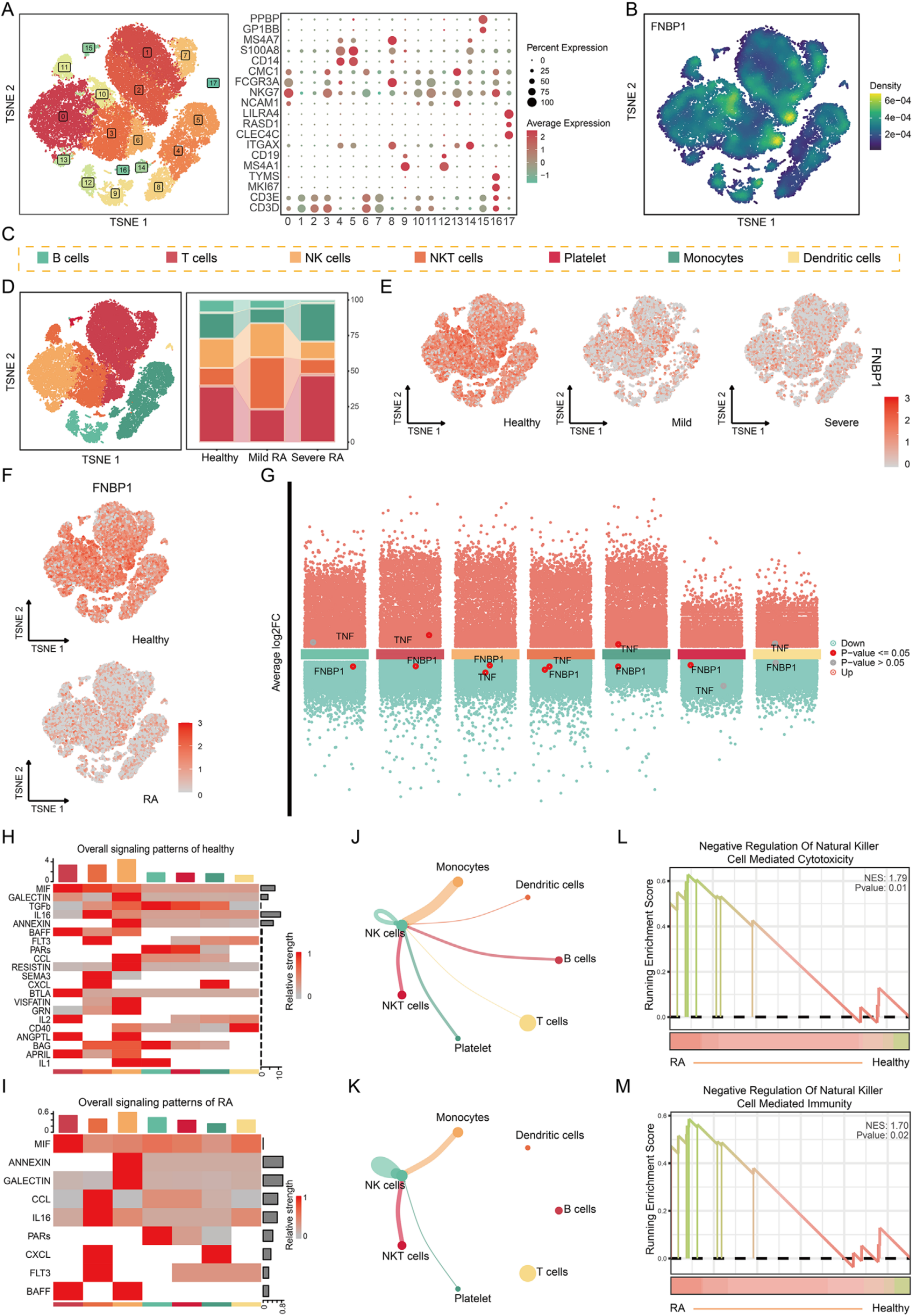
FNBP1 mediates RA progression through modulation of NK cell cytotoxicity. (A): TSNE plot of single‐cell data annotated by cell type, accompanied by marker gene expression dot plot. (B): Gene expression density map of FNBP1. (C): Color labels for seven types of immune cells. (D): Comparison of cell‐type proportions across different samples. (E): Differential expression of FNBP1 across RA severity grades and sample types. (F): Expression levels of FNBP1 in distinct immune cell subsets between RA patients and healthy controls. (G,H): Heatmaps depicting intercellular communication networks in healthy individuals (G) and RA patients (H). (I,J): Chord diagrams illustrating NK cell interactions in healthy individuals (I) and RA patients (J). (K): GSEA enrichment analysis comparing RA‐associated NK cells and NK cells from healthy individuals. (nRA Single‐cell Data = 2, nHealthy Single‐cell Data = 2).

We performed a single‐cell analysis to investigate the critical role of NK cell cytotoxic function in RA. Following dimensionality reduction, we annotated 18 distinct cell subpopulations (Figure 5A). Subsequently, based on immune cell marker genes, we identified 7 kinds of immune cells (Figure 5C,D). Gene expression profiling revealed that FNBP1 was predominantly expressed in virus‐responsive cell types, including NK cells and NKT cells (Figure 5B). Interestingly, we observed a slight increase in NK cell numbers in patients with mild RA, whereas a marked reduction was evident in those with severe RA (Figure 5D). Correspondingly, FNBP1 expression was significantly decreased in all immune cell populations from RA samples compared to healthy controls (Figure 5E,G), with a clear downward gradient from healthy to mild and then to severe RA (Figure 5E). This trend was particularly pronounced in NK cells. Cell–cell communication analysis further demonstrated a substantial reduction in the interactions between NK cells and other immune cells in RA samples relative to controls (Figure 5H–K). Moreover, key NK cell–associated cytotoxic signaling pathways (TGF‐β, CD40, and IL‐1) were notably absent in RA samples (Figure 5H–I), with this pattern also reflected across RA severity classifications (Figure S5). Finally, GSEA confirmed significant suppression of NK cell‐mediated cytotoxic and immune functions in RA (Figure 5L,M).

Taken together, these findings suggest that exposure to PM_10_ downregulates FNBP1 expression, thereby impairing NK cell cytotoxicity and contributing to the initiation and progression of RA.

### 3.5 FNBP1 Mediates Cytotoxic Functions by Regulating the Differentiation of NK Cells

**FIGURE 6:**
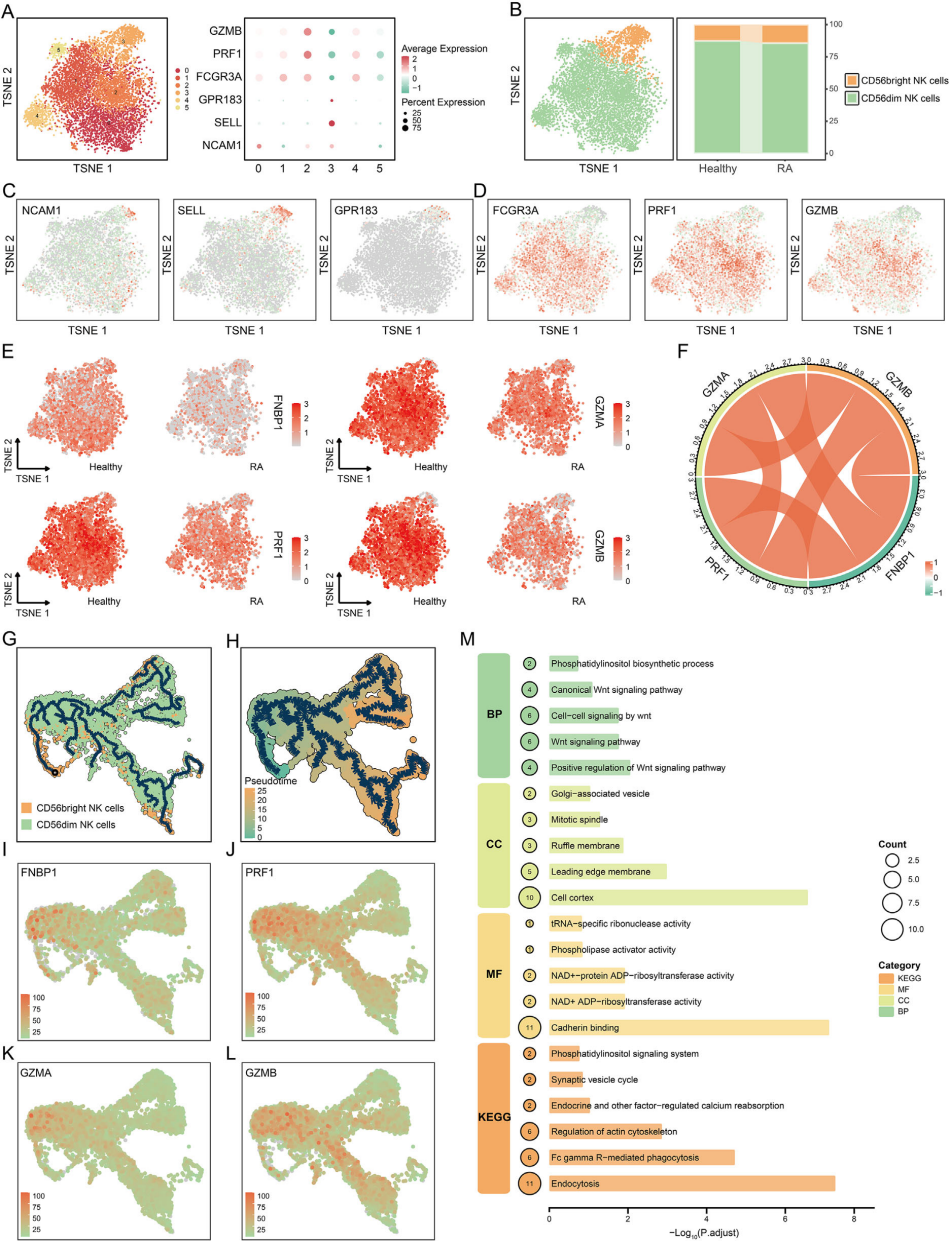
FNBP1 regulates the differentiation of CD56dim NK cells. (A): TSNE plot and marker gene dot plot showing secondary annotation of single‐cell data. (B): Annotated t‐SNE map of NK cells and comparison of cell type proportions across groups. (C,D): TSNE plots illustrating the expression patterns of marker genes in CD56^bright^ and CD56^dim^ NK cell subsets. (E): TSNE plot showing inter‐group differential expression of FNBP1 and virus‐related genes, including PRF1, GZMA, and GZMB. (F): Chord plot depicting correlations between FNBP1 and virus‐related genes. (G,H): Trajectory plots illustrating the differentiation paths of NK cells. (I–L): Dynamic changes in the expression trajectories of FNBP1 and virus‐related genes during NK cell differentiation. (M): GO and KEGG pathway enrichment analyses of FNBP1 and its interacting genes (BP, biological process; CC, cellular component; MF, molecular function). (nRA Single‐cell Data = 2, nHealthy Single‐cell Data = 2).

To further investigate the molecular role of FNBP1, we isolated NK cell subsets and conducted secondary dimensionality reduction, clustering, and annotation analyses (Figure 6A). This approach enabled the identification of two canonical NK cell subtypes: CD56^bright^ and CD56^dim^, as validated by the expression profiles of their signature genes (Figure 6C,D). These two subtypes play distinct immunological roles—CD56^bright^ NK cells are primarily involved in immune regulation and cytokine secretion, whereas CD56^dim^ NK cells exert cytotoxic effects via the secretion of perforin (PRF1) and granzymes (GZMA, GZMB) [36, 37]. In RA samples, we observed a marked reduction in CD56^dim^ NK cells (Figure 6B), consistent with our prior findings. Moreover, expression levels of FNBP1 and the cytotoxicity‐associated genes PRF1, GZMA, and GZMB were significantly downregulated in RA (Figure 6E). Notably, FNBP1 expression showed a strong positive correlation with these effector molecules (Figure 6F). Furthermore, the expression of FNBP1 is primarily concentrated in CD56^dim^ NK cells (Figure S6), which also suggests that it plays an important role in the specific cytotoxic function of this subpopulation of cells.

Our results confirm that PM_10_ exposure inhibits the differentiation of NK cells (Figure 4I) and suggest a potential role for FNBP1 in this process. To investigate this, we performed a pseudotime analysis, and the inferred trajectory suggests a potential differentiation path from CD56^bright^ to CD56^dim^ NK cells (Figure 6G,H). This finding aligns with the established biological paradigm that CD56^bright^ NK cells serve as precursors to CD56^dim^ NK cells. Moreover, during NK cell differentiation, FNBP1 expression progressively increased (Figure 6I), accompanied by a concomitant upregulation of virus‐related genes (Figure 6J–L). These observations suggest that FNBP1 may be involved in the process of NK cell differentiation and could serve as a potential driver in the onset and progression of RA induced by PM_10_ exposure.

To further elucidate the molecular interactions of FNBP1, we analyzed its PPI network using the STRING database (Figure S7) and explored the functional synergy of its interacting partners through GO and KEGG pathway analyses. Our results demonstrate that FNBP1 and its associated genes are predominantly involved in regulating the Wnt signaling pathway (Figure 6M). Previous studies have established that the Wnt pathway orchestrates NK cell development and functional maturation via coordinated regulation of canonical and non‐canonical signaling cascades, modulation of key transcription factors, and crosstalk with other signaling pathways [38]. In summary, we propose a novel mechanism whereby PM_10_ exposure downregulates FNBP1 expression, thereby disrupting Wnt signaling and impairing NK cell differentiation, ultimately contributing to the pathogenesis of RA.

### 3.6 FNBP1 Expression in the Rat Model Mirrored the Patterns Predicted by Bioinformatics Analyses

**FIGURE 7:**
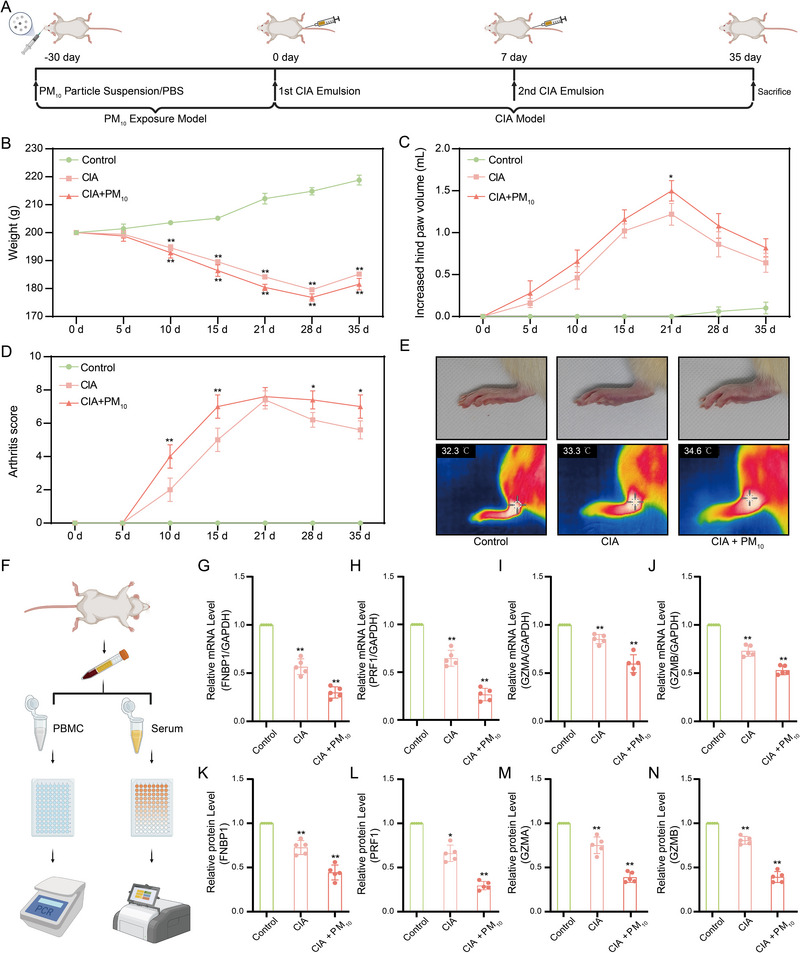
Validation of FNBP1 expression patterns in a rat model. (A): Schematic overview of the rat model construction. (B): Line graph depicting body weight changes across experimental groups. (C): Line graph showing variations in paw volume among the different groups. (D): Line graph illustrating arthritis score progression in each group. (E): Comparison of gross morphology and surface temperatures of the feet and claws among groups. (F): Workflow for peripheral blood analysis in rats. (G–J): qRT‐PCR results showing expression levels of FNBP1 and virus‐associated genes across experimental groups. (K–N): ELISA‐based quantification of FNBP1 and virus‐associated proteins in the different rat groups. (Data are expressed as mean ± SD, two‐group comparison analyzed by Wilcoxon test, and multi‐group comparison analyzed by one‐way ANOVA; ^*^ *p* < 0.05, ^**^ *p* < 0.01; in all datasets, *n* = 5 independent samples).

To validate the results of our analysis, we established a rat model exposed to PM_10_, upon which we subsequently induced CIA. The detailed experimental procedure is illustrated in Figure 7A. Our findings demonstrated that body weight increased steadily in the Control group, whereas weight gain was suppressed in both the CIA and CIA + PM_10_ groups, with a more pronounced weight loss observed in the CIA + PM_10_ group (Figure 7B). Paw swelling and arthritis scores progressively increased in the CIA group; however, the CIA + PM_10_ group exhibited a higher peak and a prolonged duration of inflammation. Additionally, joint redness, swelling, and thermal imaging temperature were markedly exacerbated by PM_10_ exposure, indicating aggravated joint inflammation in CIA rats (Figure 7C–E). PBMCs and serum were isolated from each group for qPCR and ELISA analyses of FNBP1, PRF1, GZMA, and GZMB expression (Figure 7F). Compared with Controls, the CIA group showed upregulated expression of these genes and their corresponding serum proteins. Notably, PM_10_ exposure modulated these immune‐related markers further in the CIA + PM_10_ group, synergistically exacerbating joint inflammation (Figure 7G‐N). These results provide mechanistic insights into the role of environmental factors in the pathogenesis of autoimmune arthritis.

### 3.7 PM_10_ Mediates FNBP1 to Regulate the Cytotoxicity of CD56^dim^ NK Cells

**FIGURE 8:**
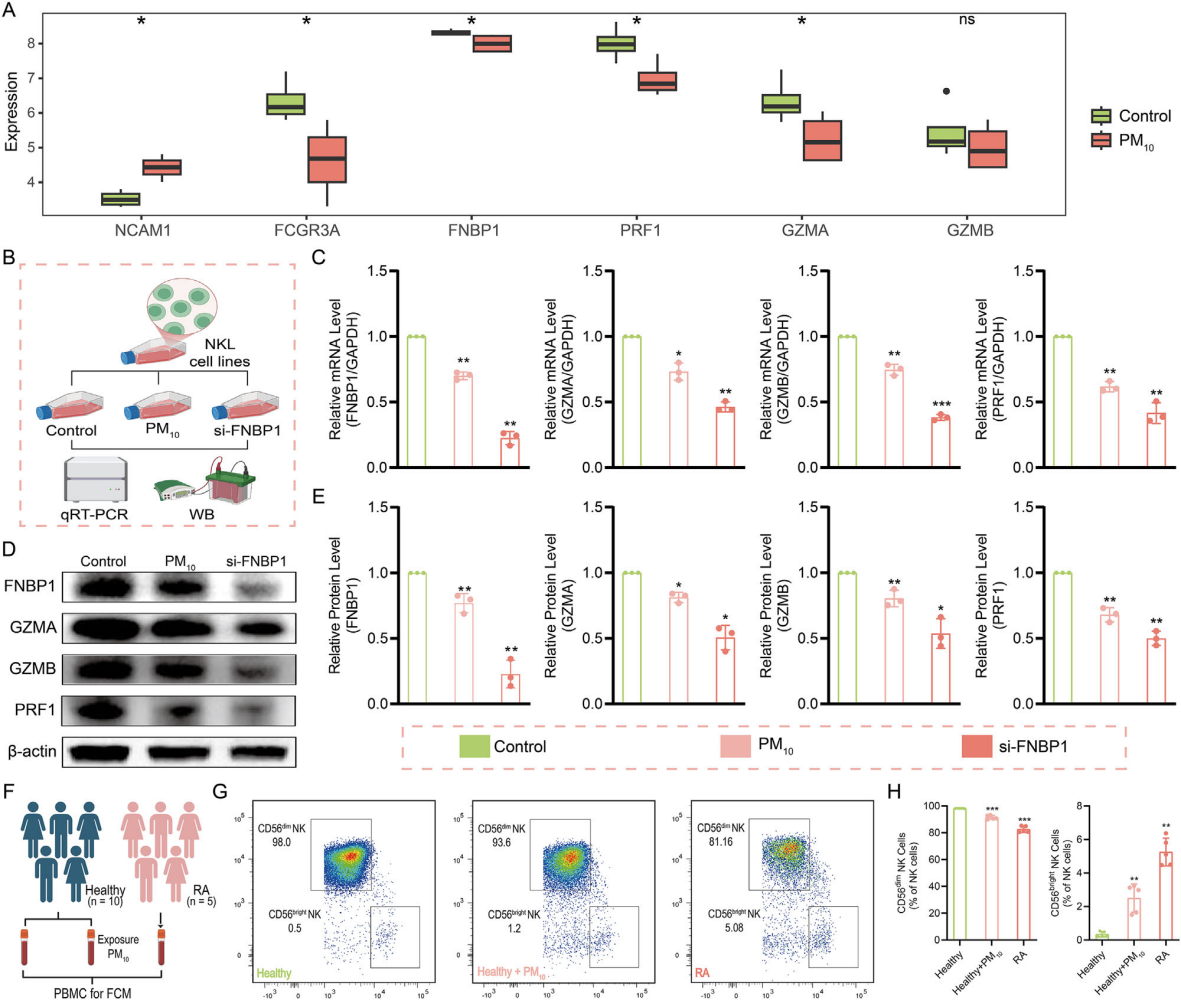
PM_10_ mediates FNBP1 downregulation and impairs the cytotoxicity of CD56^dim^ NK cells. (A): Transcriptomic data showing downregulated expression of FNBP1, PRF1, GZMA, and GZMB in PM_10_ exposed PBMC compared with healthy group. (B): Schematic of FNBP1 deficient (siRNA transfected) and PM10 exposed NKL cell models. (C–E): qRT‐PCR and Western blot analyses revealed the changes in mRNA and protein levels of FNBP1 and genes related to cytotoxicity (PRF1, GZMA, GZMB). (F): Workflow for investigating PM_10_‐FNBP1‐CD56^dim^ NK cell cytotoxicity regulation. (G): Representative dot plots of CD56^dim^/CD56^bright^ NK subsets. (H): Quantitative CD56^dim^/CD56^bright^ NK cell proportions. (Data are expressed as mean ± SD, two‐group comparison analyzed by Wilcoxon test, and multi‐group comparison analyzed by one‐way ANOVA; ns = not significant; ^*^ *p* < 0.05, ^**^ *p* < 0.01, ^***^ *p* < 0.001; nGSE226707 = 8, in NKL experiment, *n* = 3 independent samples, in flow cytometry experiment, *n* = 5 independent samples).

First, we re‐analyzed and validated PBMC transcriptomic data from individuals exposed to PM_10_. As shown in Figure 8A, PM_10_ exposed PBMC exhibited marked downregulation of FNBP1 as well as key cytotoxic effector molecules (PRF1, GZMA, and GZMB) compared with healthy controls. To further corroborate these findings, we established FNBP1‐deficient and PM_10_ exposed NKL cell models (Figure 8B). The qRT‐PCR and WB analyses confirmed a consistent reduction in FNBP1 mRNA and protein levels in both models (Figure 8C–E), indicating that PM_10_ exposure phenocopies FNBP1 deficiency and positioning FNBP1 as a downstream target of PM_10_. Correspondingly, the expression of cytotoxic effector genes (PRF1, GZMA, and GZMB) was also reduced across both models. These results reinforce our previous bioinformatic observations and provide direct transcriptomic and experimental evidence that PM_10_ exposure cooperatively suppresses the expression of FNBP1 and the cytotoxic function of NK cells.

Finally, following the framework in Figure 8F, we further examined how PM_10_ regulates FNBP1‐mediated cytotoxicity in CD56^dim^ NK cells. To characterize phenotypic changes induced by PM_10_ exposure and RA, we performed flow cytometry on PBMC from three cohorts: group 1 (healthy samples), group 2 (healthy group treated in vitro with PM_10_), and group 3 (RA patients). Results demonstrated that the proportion of CD56^dim^ NK cells decreased significantly in group 2 (91.403% ± 1.513%) and group 3 (82.577% ± 2.317%) compared with group 1 (98.152% ± 0.0069%), whereas CD56^bright^ NK cells were proportionally increased in both exposure and disease conditions (Figure 8G–H). These results align with our bioinformatic predictions and confirm that PM_10_ exposure mediates the downregulation of FNBP1 and impairs the cytotoxic phenotype of CD56^dim^ NK cells.

## 4 Discussion

In this study, we systematically integrated multi‐dimensional evidence, including epidemiological data, bioinformatics analyses, single‐cell transcriptomic sequencing, and animal experiments, to infer the potential mechanism by which airborne particulate matter suppresses FNBP1 expression, thereby impairing NK cell function and promoting the onset and progression of RA. The proposal of this scientific hypothesis regarding the “PM_10_‐FNBP1‐NK cells” axis not only expands current understanding of RA pathogenesis but also provides a novel theoretical framework for future risk stratification, environmental intervention, and the development of targeted therapeutic strategies.

Previous studies have suggested a potential association between AP and increased RA risk [39, 40]. However, most have been limited to epidemiological correlations without establishing a causal relationship. Utilizing data from the GBD database, we conducted a global trend analysis of RA prevalence and observed that since 1990, over 95% of countries have experienced an upward trajectory in RA prevalence [41, 42]. Notably, this increase was especially pronounced in countries burdened by severe air pollution, such as Albania and South Korea. MR analysis further indicated a significant positive causal relationship between PM_10_ exposure and RA risk, with the robustness of these findings supported by multiple sensitivity analyses. Collectively, these findings highlighted air pollution as a potential external risk factor in the pathogenesis of RA and emphasized the importance of exploring its underlying molecular mechanisms.

Through machine learning approaches integrating multiple algorithms, FNBP1 emerged as a representative APRG among thousands of candidates. It consistently ranked first in importance across various models and demonstrated significantly higher expression levels in peripheral blood compared to other candidates such as PTBP1, suggesting superior detectability and translational potential. Moreover, FNBP1 expression was significantly inversely correlated with both RA presence and symptom severity, indicating its potential utility not only as a biomarker for disease risk prediction but also for monitoring disease progression.

Importantly, we observed marked downregulation of FNBP1 in peripheral blood from RA patients and in transcriptomic profiles of PBMCs exposed to PM_10_, suggesting FNBP1 as a key mediator of PM_10_ induced RA pathogenesis. Immune infiltration analyses revealed a positive correlation between FNBP1 expression and CD56^dim^ NK cells abundance [43]. With significant reductions in these cells observed in RA patients alongside concomitant decreases in cytotoxic effectors such as GZMA, GZMB, and PRF1 [44, 45, 46]. GSEA indicated that PM_10_ exposure inhibits NK cell differentiation pathways, while downregulation of FNBP1 dampens NK cell–mediated cytotoxic responses. Molecular docking simulations show that the representative small molecules of PM_10_ can directly bind to the molecular pocket of FNBP1, indicating a direct mechanistic interaction between particulate matter and the downregulation of FNBP1. However, these findings are limited by the complexity of PM_10_ composition, which may involve additional molecular interactions not captured in this study.

Beyond its known roles in vesicle endocytosis and cell migration, FNBP1 plays a crucial role in NK cell differentiation [47]. Single‐cell analysis revealed a reduction in overall NK cell numbers in RA patients, particularly a pronounced decrease in the CD56^dim^ subset. Pseudotime trajectory analysis suggests a potential differentiation path from CD56^bright^ to CD56^dim^ NK cells. Along this inferred trajectory, FNBP1 expression progressively increases and shows positive correlation with the expression of cytotoxic molecules, including PRF1, GZMA, and GZMB. Animal experiments further support a close association between NK cell cytotoxic function and the severity of joint injury in rats, suggesting that FNBP1 may be a potentially important mediator in this process. Protein–protein interaction network analysis of FNBP1‐correlated genes, followed by GO and KEGG enrichment analyses, indicated that the Wnt signaling pathway may underlie FNBP1‐mediated regulation of NK cells development and functional maturation. This finding aligns with previous evidence highlighting the role of the Wnt pathway in NK cells homeostasis and responsiveness, thereby lending support to our proposed “PM_10–_FNBP1–NK cells” regulatory axis hypothesis [48]. Subsequently, we established FNBP1‐deficient and PM_10_‐exposed NKL cell models. Our findings revealed that PM_10_ exposure elicited effects highly similar to those of FNBP1 impairment, thus verifying FNBP1 as a downstream target of PM_10_ to a certain extent. In both models, expression of cytotoxicity‐related genes (PRF1, GZMA, and GZMB) was reduced. Finally, flow cytometric analysis revealed a decreased proportion of CD56^dim^ NK cells in both PM_10_ exposed and RA groups compared with the control group. These results were consistent with bioinformatic predictions and collectively suggest that PM_10_ exposure may impair the cytotoxic phenotype of CD56^dim^ NK cells by downregulating FNBP1.

Epidemiological studies have shown that environmental AP (including PM_10_ and certain gaseous pollutants) is associated with an increased risk of autoimmune diseases such as RA. Large‐scale cohort studies, such as the UK Biobank and various reviews, have confirmed that long‐term combined exposure is positively correlated with RA risk, with this association being more pronounced in populations with high genetic susceptibility [49]. Mechanistically, particulate matter exposure can alter immune profiles, activate oxidative stress and pro‐inflammatory pathways, providing a biological basis for the association between the two [50]. However, differences in study models, doses, and pollutant compositions add complexity to cross‐study comparisons. The main innovation of this study lies in integrating multi‐omics data and employing multimodal approaches to establish a comprehensive evidence framework linking environmental pollution with the pathogenesis of RA, successfully unveiling a mechanistic hypothesis of RA, the “PM_10_‐FNBP1‐NK cells” axis.

However, this study has several limitations. First, the transcriptomic and epidemiological datasets primarily use cross‐sectional designs, which limit the ability to infer causal relationships and conduct dynamic temporal analyses of disease progression. Future research should involve longitudinal cohort studies, real‐time pollution monitoring, and immune dynamics analysis to more clearly establish causal relationships. Second, the sample size of public databases is limited, and there is insufficient population diversity, with a tendency to focus on specific geographic regions or ethnic groups, mainly from Europe, Asia (Japan), and Southwest China. There is a lack of validation from other regions of China (e.g., North and South China) and African populations, which limits the generalizability of the conclusions. Extensive validation across multiple centers and diverse populations is urgently needed. Third, although animal models provide mechanistic insights, the rats particulate matter exposure model established by tracheal instillation, while allowing precise control of PM_10_ dosage and direct delivery to the lungs, significantly differs from actual long‐term, low‐dose inhalation exposure in humans. Therefore, caution is needed when extrapolating localized lung tissue damage and downstream molecular signals observed in this model to clinical applications. Future studies should design experiments that better simulate human exposure scenarios.

## 5 Conclusion

By systematically integrating multi‐dimensional data, this study proposes that airborne pollutants such as PM_10_ may promote the onset and progression of RA by downregulating FNBP1 expression and consequently impairing NK cells function. FNBP1 emerges as a potential regulatory factor with considerable clinical translational value for disease monitoring and intervention. Mechanistically, PM_10_ exposure appears to disrupt immune surveillance by suppressing FNBP1, thereby exacerbating synovial inflammation and joint damage. These findings underscore the pivotal role of environmental pollutants in the pathogenesis of autoimmune disorders, providing a scientific rationale for environmental health interventions and the development of personalized therapeutic strategies.

## Author Contributions

Runhan Zhao, Qinyang Zhang and Yu Jiang: conceptualization. Runhan Zhao: formal analysis and Funding acquisition. Qinyang Zhang – original draft; Yu Jiang: data curation. Dagang Tang: investigation; Xiao Qu and Jun Zhang methodology. Weixia Duan and Tao Li: methodology and formal analysis. Yanran Huang, Zhengwei Cai and Xiaoji Luo: supervision. Yanran Huang, Zhengwei Cai and Xiaoji Luo: writing – review and editing. All authors contributed to the article and approved the final manuscript.

## Funding

This research was supported by the Doctoral Innovation Project of the First Affiliated Hospital of Chongqing Medical University (Grant: CYYY‐BSYJSKYCXXM202446).

## Ethics Statement

In this study, the collection of human peripheral blood samples was approved by the Ethics Committee of the First Affiliated Hospital of Chongqing Medical University (Approval No. 2024‐080‐01). The animal experimental protocol was reviewed and approved by the Ethics Committee of Chongqing Medical University (Approval No. IACUC‐CQMU‐2025‐06078). All experiments were conducted in strict accordance with the Declaration of Helsinki and relevant Chinese regulations on the welfare and ethics of laboratory animals.

## Conflicts of Interest

The authors declare no conflicts of interest.

## Supporting information




**Supporting File 1**: advs74082‐sup‐0001‐SuppMat.docx.

**Supporting File 2**: advs74082‐sup‐0002‐Tables.xlsx.

## Data Availability

The data that support the findings of this study are available from the corresponding author upon reasonable request.
