# Supplementary material for: PM10 Impairs CD56dim NK Cell Cytotoxicity via FNBP1 Suppression to Exacerbate Rheumatoid Arthritis: Insights from Multimodal Multi‐Omics
Source: Adv Sci (Weinh). 2026 Feb 20;13(19):e14260. doi: 10.1002/advs.202514260 (PMC13045476; doi:10.1002/advs.202514260)
Supplement: Supplementary file 1 — Supporting File 1: advs74082‐sup‐0001‐SuppMat.docx. [file ADVS-13-e14260-s001.docx]

**
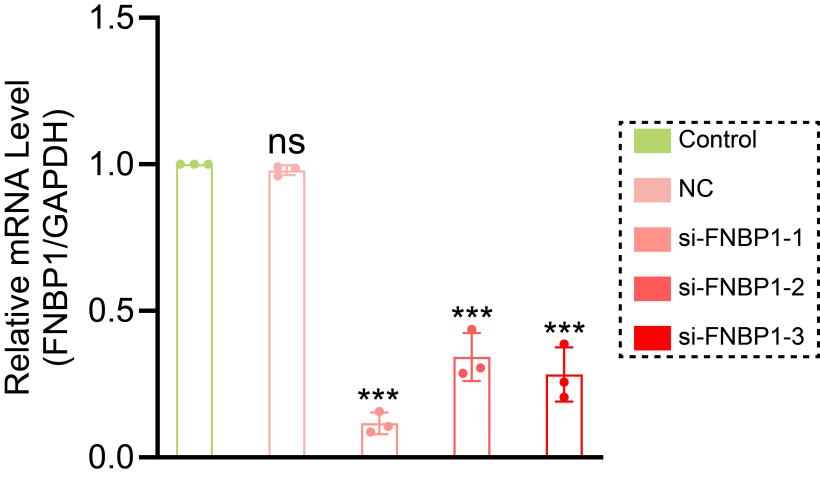
**

**Supplementary Figure 1 | The effects of three si-FNBP1 sequences on the expression level of FNBP1.** (Data are expressed as mean ± SD, multi-group comparison analyzed by one-way ANOVA; ns = not significant; ***P < 0.001; n = 3 ).

**
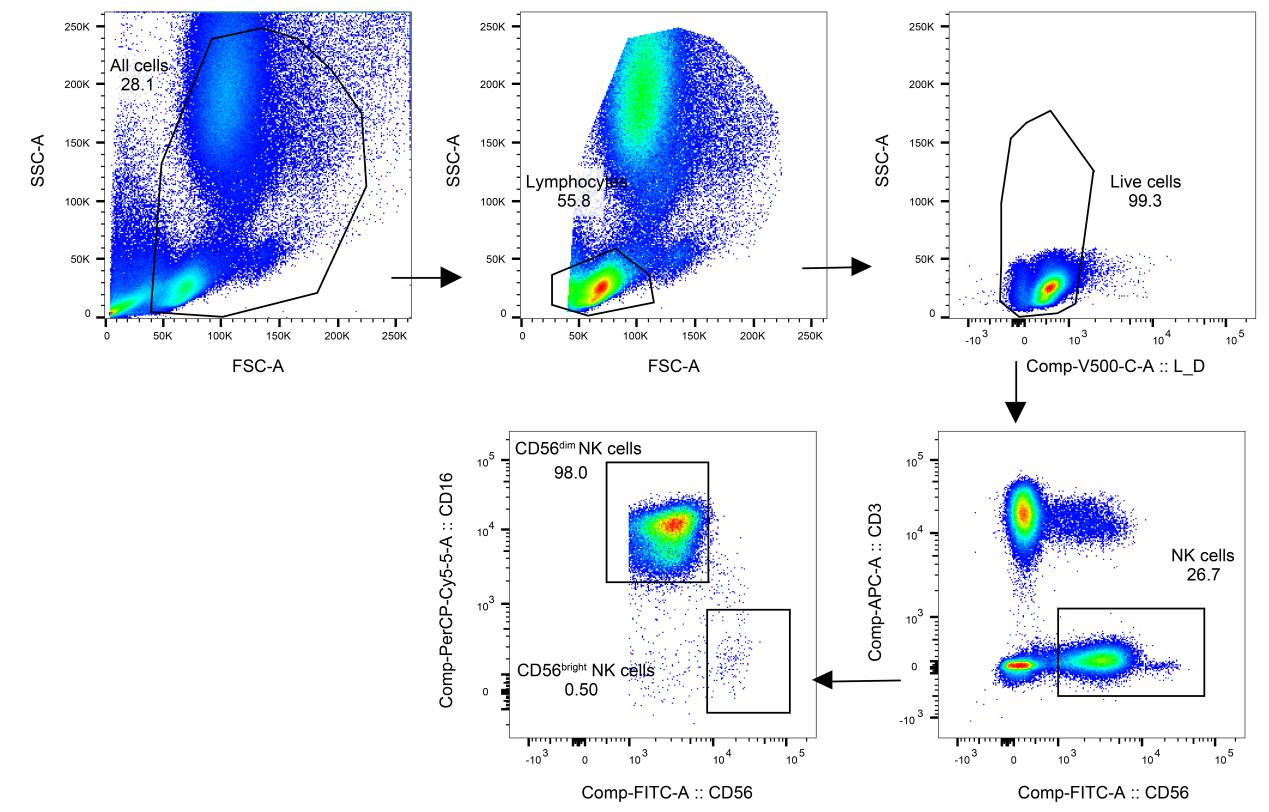
**

**Supplementary Figure 2 | The analysis strategy of human PBMC by flow cytometry.**


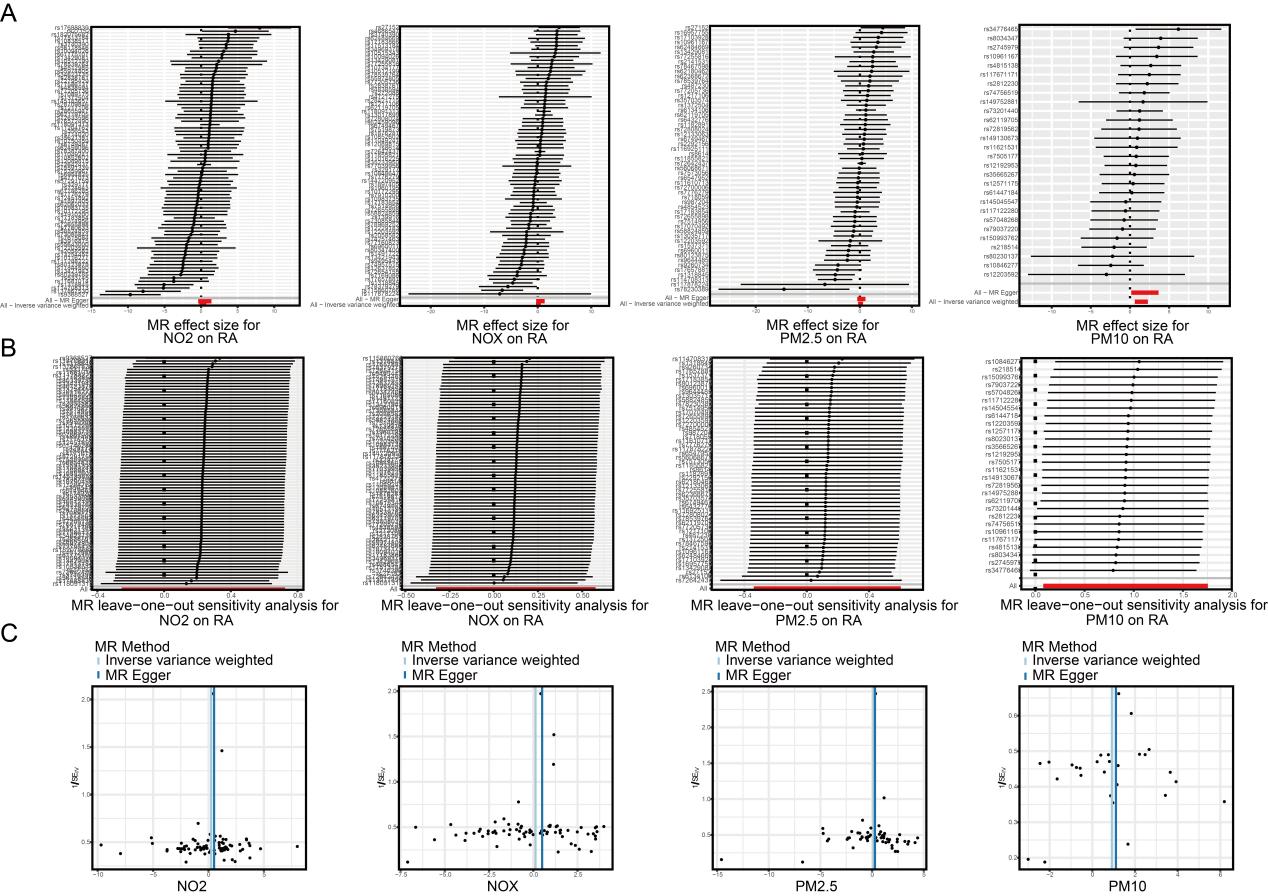


**Supplementary Figure 3 | Sensitivity analyses of the Mendelian randomization results assessing the association between AP and RA.**
**A:** Forest plot depicting the causal effect estimates of individual SNPs on RA risk. **B:** Leave-one-out analysis illustrating the influence of excluding each SNP on the overall RA outcome. **C:** Funnel plot evaluating potential outlier SNPs, used as instrumental variables.


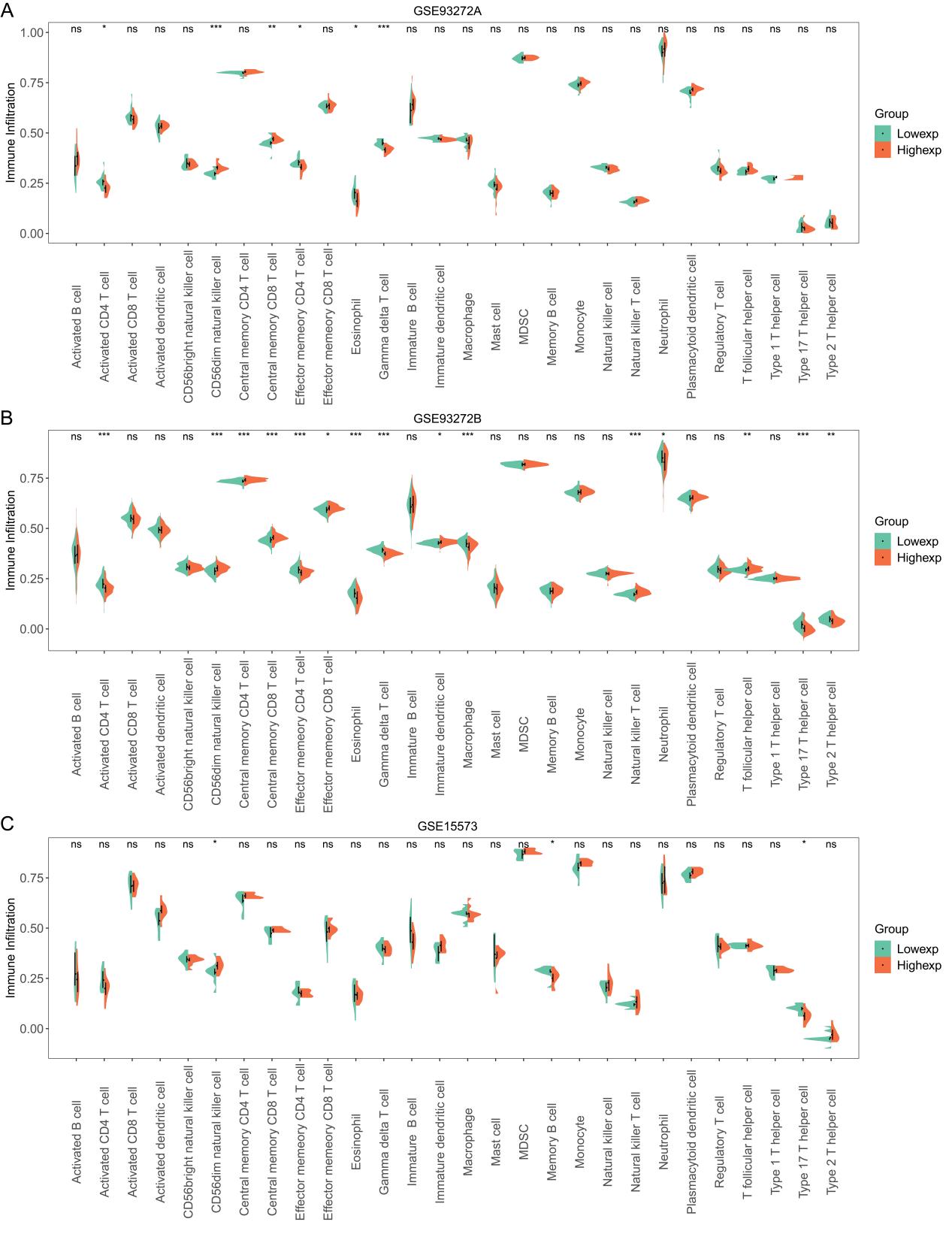


**Supplementary Figure 4 | Box plots illustrating differences in immune cell infiltration stratified by FNBP1 expression levels.**
**A–C:** Immune cell infiltration results from datasets GSE93272A, GSE93272B, and GSE15573, respectively. (Data are expressed as mean ± SD, two-group comparison analyzed by Wilcoxon test, ns = not significant; *P < 0.05, **P < 0.01, ***P < 0.001; nGSE93272A = 60, nGSE93272B = 215, and nGSE15573 = 33).


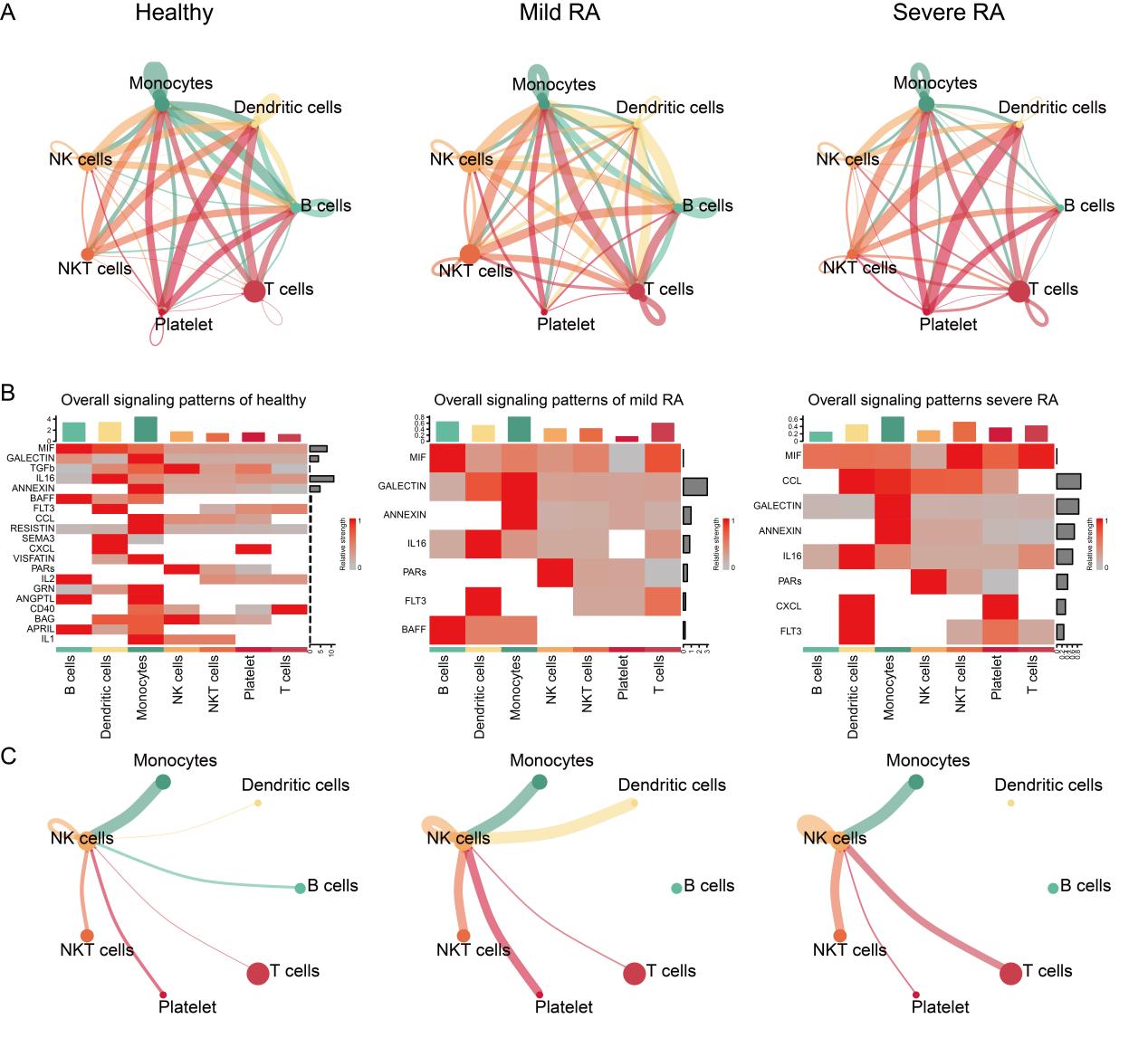


**Supplementary Figure 5 | Cell–cell communication analysis stratified by disease severity.**
**A:** Network diagrams illustrating cell–cell communication patterns across different sample groups. **B:** Heatmaps showing the relative intensity of intercellular signaling among the groups. **C:** Chord diagrams depicting the regulatory interactions of NK cells in each sample.


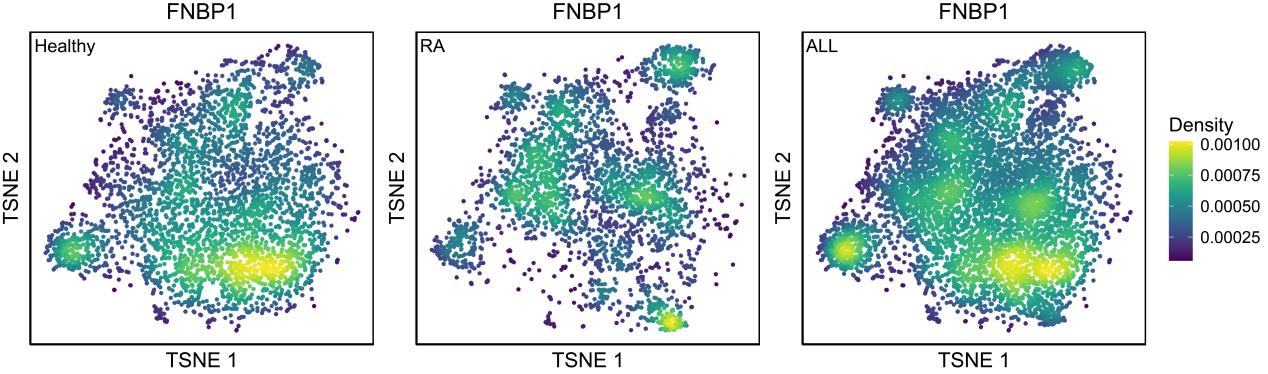


**Supplementary Figure 6 | The expression of FNBP1 in NK cells.**


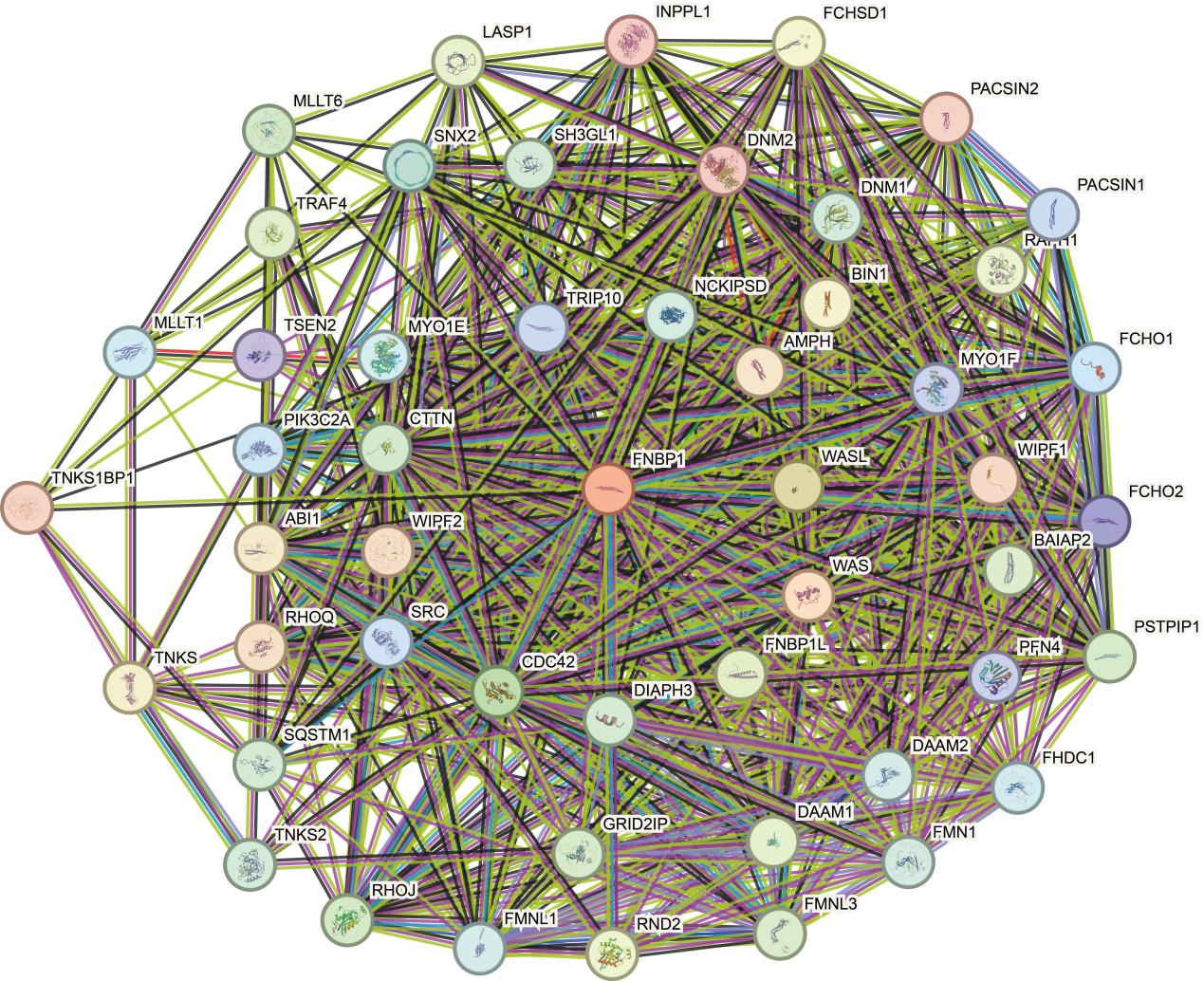


**Supplementary Figure 7 | PPI network of FNBP1.**

**Supplementary Table 1 | APRGs list from CTD database.**

**Supplementary Table 2 | Information on datasets included in this study.**

**Supplementary Table 3 | The primers of qRT-PCR in the study.**

**Supplementary Table 4 | EPAC ranking of ra in the world.**

**Supplementary Table 5 | Mendelian randomization results for AP and RA.**

**Supplementary Table 6 | Candidate key APRGs identified by univariate logistic regression algorithm.**

**Supplementary Table 7 | All prediction models based on integrated machine learning.**

**Supplementary Table 8 | Correlation analysis statistics summary table.**

**Supplementary Table 9 | PM_10_ ID annotation table.**
